# Selective Catalytic Combustion of Hydrogen under Aerobic Conditions on Na_2_WO_4_/SiO_2_

**DOI:** 10.1002/anie.202412932

**Published:** 2024-10-29

**Authors:** Elijah R. Kipp, Javier Garcia‐Barriocanal, Aditya Bhan

**Affiliations:** ^1^ Department of Chemical Engineering and Materials Science University of Minnesota Twin Cities 421 Washington Ave. SE 55455 Minneapolis Minnesota USA; ^2^ Characterization Facility University of Minnesota Twin Cities 100 Union St. SE 55455 Minneapolis Minnesota USA

**Keywords:** heterogeneous catalysis, kinetics, selective hydrogen combustion, selective oxidation, X-ray absorption spectroscopy

## Abstract

Na_2_WO_4_/SiO_2_, a material known to catalyze alkane selective oxidation including the oxidative coupling of methane (OCM), is demonstrated to catalyze selective hydrogen combustion (SHC) with >97 % selectivity in mixtures with several hydrocarbons (CH_4_, C_2_H_6_, C_2_H_4_, C_3_H_6_, C_6_H_6_) in the presence of gas‐phase dioxygen at 883–983 K. Hydrogen combustion rates exhibit a near‐first‐order dependence on H_2_ partial pressure and are zero‐order in H_2_O and O_2_ partial pressures. Mechanistic studies at 923 K using isotopically‐labeled reagents demonstrate the kinetic relevance of H−H dissociation and absence of O‐atom recombination. In situ X‐ray diffraction (XRD) and W L_III_‐edge X‐ray absorption spectroscopy (XAS) studies demonstrate, respectively, a loss of Na_2_WO_4_ crystallinity and lack of second‐shell coordination with respect to W^6+^ cations below 923 K; benchmark experiments show that alkali cations must be present for the material to be selective for hydrogen combustion, but that materials containing Na alone have much lower combustion rates (per gram Na) than those containing Na and W. These data suggest a synergy between Na and W in a disordered phase at temperatures below the bulk melting point of Na_2_WO_4_ (971 K) during SHC catalysis. The Na_2_WO_4_/SiO_2_ SHC catalyst maintains stable combustion rates at temperatures ca. 100 K higher than redox‐active SHC catalysts and could potentially enable enhanced olefin yields in tandem operation of reactors combining alkane dehydrogenation with SHC processes.

## Graphical abstract

Na_2_WO_4_/SiO_2_, a formulation known to be active for the oxidative coupling of methane (OCM), catalyzes selective hydrogen combustion (SHC) in H_2_‐hydrocarbon‐O_2_ mixtures at 873–983 K, with H_2_ combustion kinetics dependent only on the hydrogen partial pressure. X‐ray absorption spectroscopy and X‐ray diffraction demonstrate that the Na_2_WO_4_ phase is disordered under reaction conditions, even at temperatures below the bulk Na_2_WO_4_ melting point.

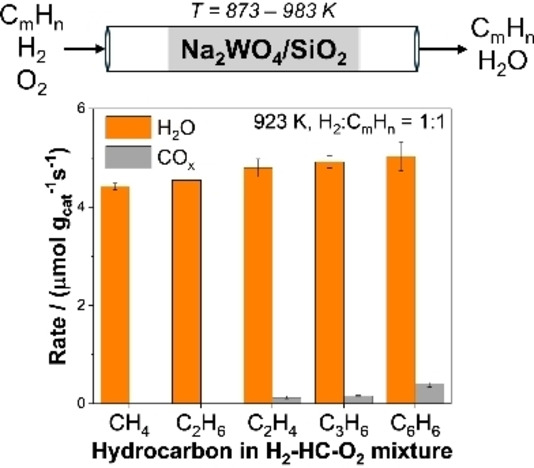

Ethylene and propylene are produced industrially primarily through endothermic and equilibrium‐limited dehydrogenation reactions. The state‐of‐the‐art process for their production, steam cracking, accounts for 8 % of the total energy demands in the chemical sector^[1]^ and nearly 1 % of total global CO_2_ emissions.^[2]^ While oxidative conversion routes can remove thermodynamic limitations associated with alkane dehydrogenation, direct oxidative dehydrogenation (ODH) processes over transition metal oxide formulations have to‐date failed to realize the necessary selectivity and catalyst stability required for industrial application.^[3]^ An alternative oxidative route for olefin production couples endothermic dehydrogenation (DH) and exothermic selective hydrogen combustion (SHC) steps to facilitate autothermal operation and circumvent single‐pass equilibrium conversion thresholds, as shown in Scheme 1;[^4^, ^5^, ^6^] the SHC step requires a catalyst which is able to preferentially oxidize hydrogen over other hydrocarbons (e.g., C_2_H_4_ and C_2_H_6_) which would be present in the effluent stream of a DH reaction.

**Scheme 1:**
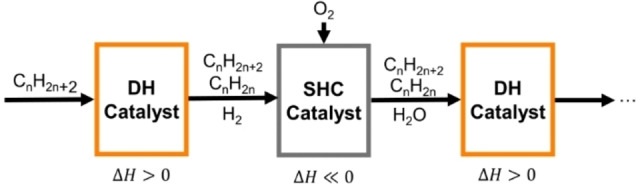
Schematic of a sequential dehydrogenation (DH)+selective hydrogen combustion (SHC) process with DH and SHC catalysts arranged in series and O_2_ cofed to the SHC reactor. Adapted from Grasselli et al.^[6]^

While SHC processes in chemical looping (CL) mode (i.e., explicitly separating reduction and oxidation half‐cycles and using lattice oxygen as the oxidant for hydrogen combustion) have been reported,[^5^, ^7^, ^8^, ^9^, ^10^] aerobic SHC processes (in which O_2_ is cofed in sub‐stoichiometric amounts relative to H_2_) offer several advantages over CL‐SHC by enabling continuous operation and eliminating the need for external heating of the SHC reactor (which would otherwise be required during endothermic reduction half‐cycles).

We report herein that 5 wt % Na_2_WO_4_/SiO_2_, a formulation which is also known to catalyze the oxidative coupling of methane (OCM, 2 CH_4_+1/2 O_2_→C_2_H_6_+H_2_O),[^11^, ^12^, ^13^, ^14^] can preferentially combust hydrogen in equimolar mixtures with several other hydrocarbons in the presence of gas‐phase dioxygen at 903–983 K. Consistent with the high stability of Na_2_WO_4_‐based catalysts observed during OCM,^[15]^ Na_2_WO_4_/SiO_2_ remains active for hydrogen combustion with negligible change in rates over 64 h on‐stream in CH_4_−H_2_−O_2_ mixtures at 923–983 K, as shown in Figure 1a. The Na_2_WO_4_/SiO_2_ formulation is representative of a new class of alkali metal‐based aerobic SHC catalysts for which gas‐phase O_2_ generates H_2_‐selective surface oxygen species in the absence of a redox‐active support; the stability and structural disorder of Na_2_WO_4_/SiO_2_ at high operating temperatures contrasts this material with redox‐active ordered bulk oxide materials (e.g., In_2_O_3_, Bi_2_O_3_) that use lattice oxygen to catalyze the selective combustion of hydrogen.

**Figure 1:**
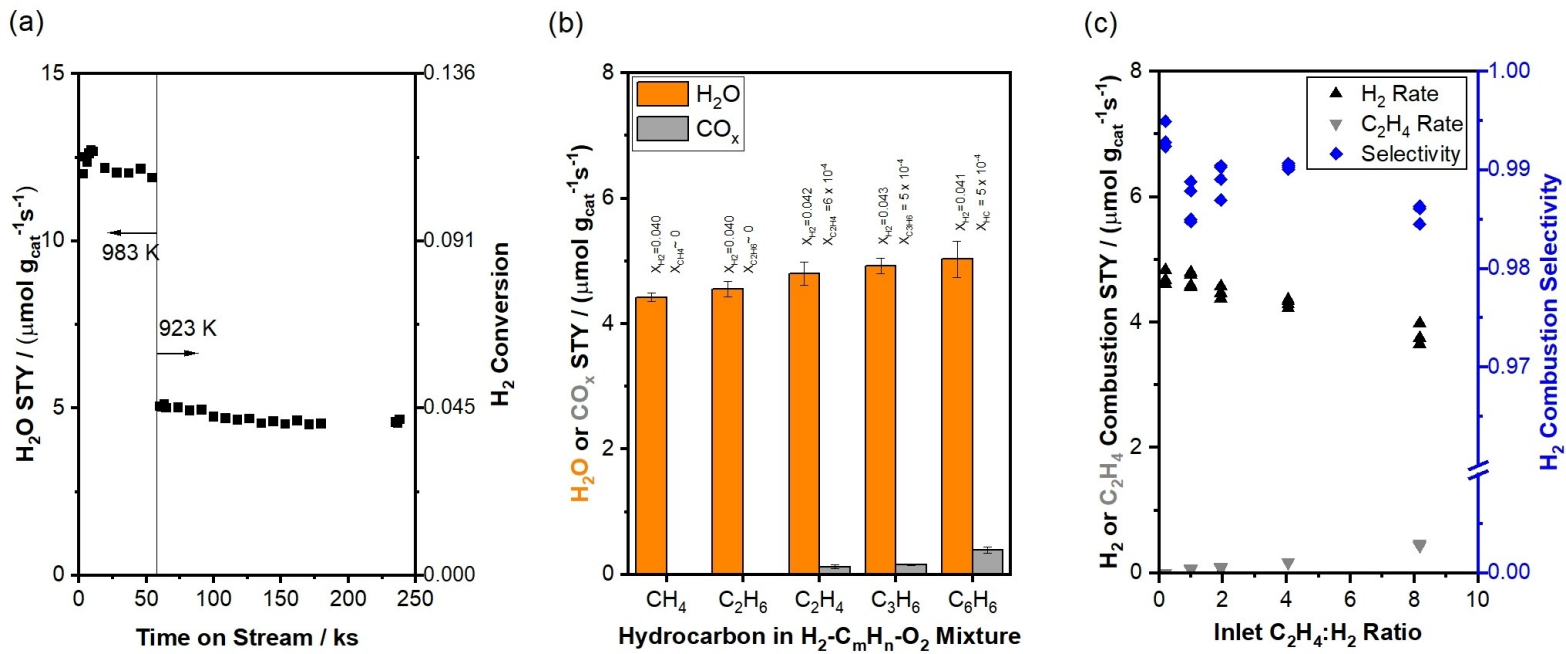
(a) H_2_O space‐time yield (STY) as a function of time on stream during SHC in CH_4_−H_2_−O_2_ mixtures over a 5 wt % Na_2_WO_4_/SiO_2_ catalyst (5 kPa CH_4_, 5 kPa H_2_, 1.25 kPa O_2_, balance He+N_2_, 1.67 cm^3^ (STP) s^−1^, 0.0290 g Na_2_WO_4_/SiO_2_). CO_x_ or C_2_ products did not form in measurable quantities at any time. H_2_ conversions are given on the right y‐axis. (b) H_2_O and CO_x_ (*x*=1,2) STYs for different H_2_‐hydrocarbon‐O_2_ mixtures (5 kPa hydrocarbon, 5 kPa H_2_, 1.25 kPa O_2_, balance He+N_2_, 1.67 cm^3^ (STP) s^−1^ total flow rate, 923 K, 0.0290 g Na_2_WO_4_/SiO_2_). Corresponding values for H_2_ conversions and C_m_H_n_ conversions (where m is the carbon number of the hydrocarbon) are given above each bar. (c) STYs associated with C_2_H_4_ and H_2_ combustion and H_2_ combustion selectivity at varying C_2_H_4_ : O_2_ molar ratios during SHC in C_2_H_4_−H_2_−O_2_ mixtures (1–41 kPa C_2_H_4_, 5 kPa H_2_, 1.25 kPa O_2_, balance He+N_2_, 1.67 cm^3^ (STP) s^−1^, 923 K, 0.0290 g Na_2_WO_4_/SiO_2_).

Experimental methods for catalyst synthesis (adapted from Kiani et al.[^14^, ^16^]), catalytic reaction experiments, and materials characterization are given in Section S1 of the Supporting Information. For binary H_2_−C_m_H_n_ mixtures, the hydrogen combustion selectivity (S_H2_) is defined according to Eq. 1:

(1) $$S_{H_{2}}=\frac{X_{H_{2}}}{X_{H_{2}}+X_{C_{m}H_{n}}}\;$$

where X_H2_ and X_CmHn_ are the hydrogen and hydrocarbon conversions due to combustion only (see Supporting Information, Section S4 for details). Figure 1b shows formation rates of H_2_O and CO_x_ in equimolar mixtures of hydrogen with CH_4_, C_2_H_4_, C_2_H_6_, C_3_H_6_, and C_6_H_6_ (H_2_ : C_m_H_n_ : O_2_=4 : 4 : 1) at 923 K. The hydrogen combustion selectivity exceeds 97 % for all H_2_−C_m_H_n_−O_2_ mixtures at the process conditions studied. For H_2_−C_2_H_4_−O_2_ mixtures, >97 % hydrogen combustion selectivities persist over 40× changes in H_2_ : C_2_H_4_ ratio at fixed H_2_ : O_2_ (Figure 1c), as well as over 10× changes in H_2_ : O_2_ ratio at fixed H_2_ : C_2_H_4_ (Figure S5). These data demonstrate that Na_2_WO_4_/SiO_2_ catalyzes selective combustion of hydrogen in mixtures with hydrocarbons over a broad range of process conditions. Given that hydrogen combustion rates are invariant with conversion (up to X_O2_=0.20, Figure S6) and with O_2_ pressure (Figure 2a), high hydrogen combustion selectivity is anticipated to persist over Na_2_WO_4_/SiO_2_ even at integral conversions and low H_2_ : C_m_H_n_ ratios.

**Figure 2:**
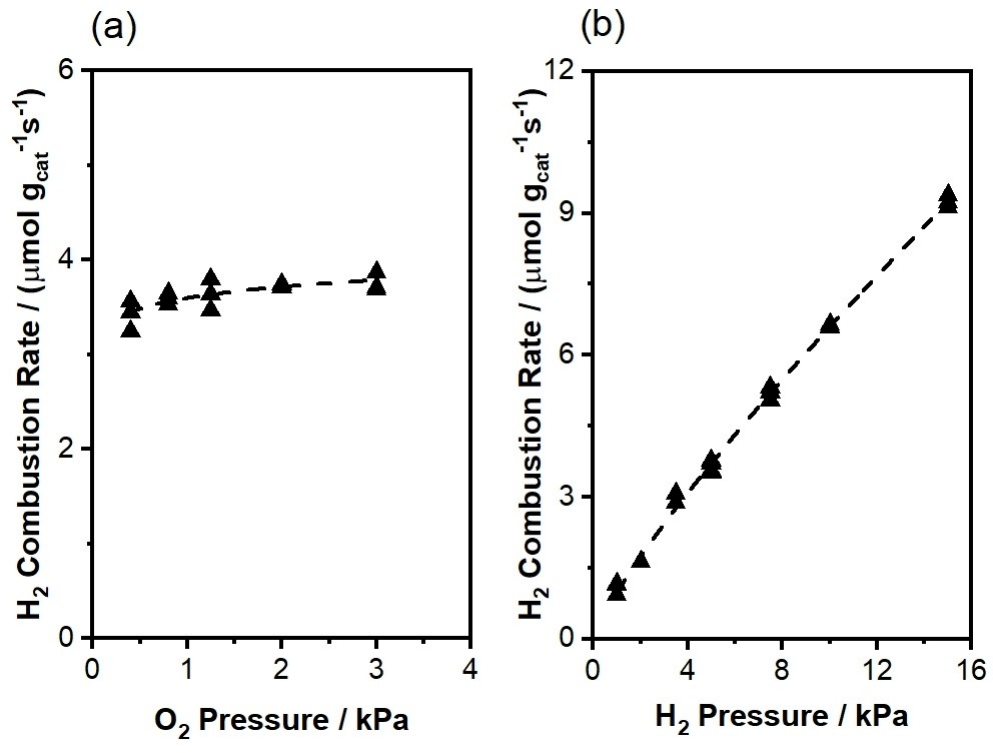
Variation of H_2_ combustion rates with (a) O_2_ partial pressure (5 kPa CH_4_, 5 kPa H_2_, balance He+N_2_, 3.33 cm^3^ (STP) s^−1^, 923 K, 0.0310 g Na_2_WO_4_/SiO_2_) and (b) H_2_ partial pressure (5 kPa CH_4_, 1.25 kPa O_2_, balance He+N_2_, 3.33 cm^3^ (STP) s^−1^, 923 K, 0.0310 g Na_2_WO_4_/SiO_2_). Apparent orders with respect to H_2_ and O_2_ partial pressures are 0.83±0.03 and 0.06±0.02, respectively.

Kinetic studies over the Na_2_WO_4_/SiO_2_ catalyst were conducted for H_2_−CH_4_−O_2_ mixtures at 923 K. No CH_4_ consumption or CO_x_ formation was detected at these conditions, and H_2_O was the only measurable reaction product. Measured SHC rates are not convoluted by gradients in concentration (Tables S1 and S3) or temperature (Tables S2, S4, and S5), as shown in Section S2 of the Supporting Information; these results were validated by experiments showing that changes in particle size (Table S7) or bed dilution (Table S8) cause negligible changes in measured H_2_O formation rates. Combustion rates in reactor beds containing only a sand diluent are insignificant at 923–983 K (Table S6) compared to combustion rates measured in the presence of Na_2_WO_4_/SiO_2_, and variations in sand loading led to negligible changes in rates (Table S8); thus, H_2_O formation rates reflect combustion reactions initiated at the Na_2_WO_4_/SiO_2_ surface rather than reactions initiated homogeneously or on other solid surfaces.

Figure 2 shows a near‐first‐order dependence of H_2_ combustion rate on H_2_ partial pressure (1–15 kPa H_2_) and zero‐order dependence on O_2_ partial pressure (0.4–3 kPa O_2_) at 923 K, suggesting that hydrogen activation is a kinetically relevant step during SHC. The measured H_2_/D_2_ kinetic isotope effect (KIE) of 1.3 (Table 1) demonstrates that cleaving H−H bonds is rate‐determining. Steady‐state ^16^O_2_−^18^O_2_ cofeed experiments (Figure 3) result in negligible ^16^O^18^O formation rates at 923 K in both the absence and the presence of H_2_, evincing that direct O_2_ dissociative adsorption is not quasi‐equilibrated during H_2_ combustion, in contrast with mechanisms proposed for OCM at higher temperatures.[^11^, ^17^, ^18^, ^19^] Instead, direct O_2_ dissociation is either irreversible (see discussion in Section S6.3 of the Supporting Information) or does not occur at a significant rate at 923 K. Na_2_WO_4_‐promoted redox‐active oxides (e.g., Na_2_WO_4_/Mg_6_MnO_8_, Na_2_WO_4_/MnO_x_) have been previously studied as anaerobic CL‐SHC catalysts by Li and co‐workers[^10^, ^20^] and by Qin et al.;^[21]^ the Na_2_WO_4_ phase has been shown via ^18^O_2_ pulse experiments to inhibit oxygen exchange with redox‐active supports. That is, O_2_ does not readily exchange with lattice oxygen species from either Na_2_WO_4_ or the underlying support.^[10]^ The absence of ^16^O^18^O formation shown in Figure 3 is consistent with this observation, demonstrating that the kinetics of O_2_ exchange over Na_2_WO_4_/SiO_2_ with both lattice oxygen species and surface oxygen species derived from gas‐phase O_2_ are significantly slower than the kinetics of H_2_ combustion.

**Table 1:** Combustion product formation rates over 5 wt % Na_2_WO_4_/SiO_2_ using different hydrogen isotopologues (5 kPa CH_4_, 5 kPa H_2_ or D_2_, 1.25 kPa O_2_, balance He, 923 K, 3.33 cm^3^ (STP) s^−1^, 0.0310 g Na_2_WO_4_/SiO_2_).

Isotope	H_2_O Rate/ (μmol g_cat_^−1^ s^−1^)	CO_x_ Rate/ (μmol g_cat_^−1^ s^−1^)	Kinetic isotope effect
H_2_	4.1±0.2	~0	1.3±0.1
D_2_	3.1±0.1	~0	–

**Figure 3:**
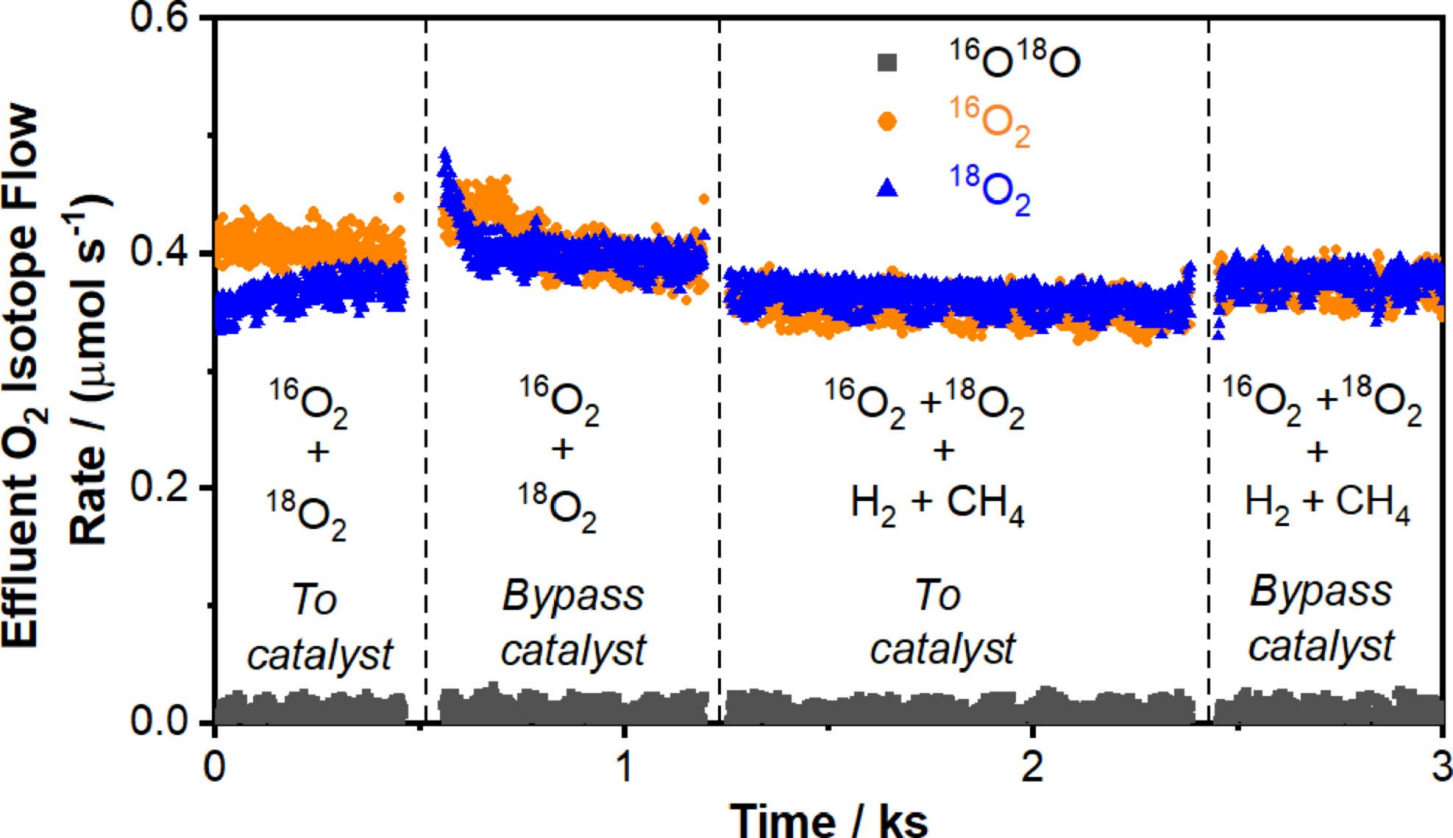
Oxygen isotope effluent flow rates during ^16^O_2_−^18^O_2_ cofeed experiments in the absence or presence of H_2_ and CH_4_ (0 or 5 kPa CH_4_, 0 or 5 kPa H_2_, 0.6 kPa ^16^O_2_, 0.6 kPa ^18^O_2_, balance He+Ar, 1.67 cm^3^ (STP) s^−1^, 923 K, 0.0310 g Na_2_WO_4_/SiO_2_). Effluent H_2_O flow rates were 0.14 μmol s^−1^ when H_2_, CH_4_, ^16^O_2_, and ^18^O_2_ were cofed to the reactor.

H_2_ combustion rates are invariant with contact time and effluent H_2_O partial pressure in mixtures with C_2_H_4_ (Figure S6), implying that reaction pathways involving the H_2_O product are insignificant during SHC. Prior OCM studies on Na_2_WO_4_/SiO_2_ and Mn/Na_2_WO_4_/SiO_2_ catalysts[^11^, ^12^, ^17^, ^18^] have shown that CH_4_ coupling rates increase with increasing H_2_O partial pressure, an effect proposed to originate from the formation of hydroxyl radicals through a pathway involving H_2_O and O_x_* species (e.g., Na_2_O_2_+H_2_O→Na_2_O+H_2_O_2_, H_2_O_2_→2 OH⋅)[^11^, ^19^] or from the formation of a more active O* species formed by the reaction of H_2_O with O_2_*.^[22]^ During SHC, we propose that H_2_ scavenges active O* or O_2_* species at a much greater rate than H_2_O, such that H_2_O‐mediated contributions are negligible under the conditions studied. The reaction enthalpy of H_2_ reacting with an O_x_* (*x*=1,2) species to form H_2_O+O_x−1_* species is 353 kJ mol^−1^ more exothermic than the reaction of H_2_O with the same O_x_* to form H_2_O_2_+O_x−1_*. For example, if Na_2_O_2_ is assumed to provide the active oxygen species for H_2_O or H_2_, as suggested by Takanabe and co‐workers,[^11^, ^19^] the standard reaction enthalpy of Na_2_O_2_+H_2_→Na_2_O+H_2_O (923 K, 1 bar) is −156 kJ mol^−1^, while the reaction enthalpy of Na_2_O_2_+H_2_O→Na_2_O+H_2_O_2_ is 197 kJ mol^−1^. H_2_O‐mediated pathways are thus expected to be insignificant with competing H_2_ pathways present.

The high hydrogen combustion selectivities shown in Figure 1b are inconsistent with radical‐based homolytic R−H bond scission mechanisms, commonly invoked to describe OCM,[^17^, ^23^, ^24^, ^25^, ^26^, ^27^] for which activation barriers are expected to scale linearly with bond dissociation energy (BDE) according to Brønsted‐Evans‐Polanyi relations. Here, high hydrogen selectivity is observed even though the H−H BDE of H_2_ (436 kJ mol^−1^) is similar to the C−H BDE of CH_4_ (439 kJ mol^−1^) and 60 kJ mol^−1^ greater than the weakest C−H BDE of C_3_H_6_ (376 kJ mol^−1^).^[28]^ An alternative descriptor related to the ease of heterolytic R−H scission over metal oxides,[^29^, ^30^] the Brønsted acidity of the weakest C−H or H−H bond, is also inadequate, as differences in deprotonation energy (DPE) also fail to describe why H_2_ (1675 kJ mol^−1^) is strongly favored for combustion versus C_6_H_6_ (1679 kJ mol^−1^) or C_3_H_6_ (1620 kJ mol^−1^). Recent computational studies on the scission of R−H bonds in hydrogen[^9^, ^31^] and other alkanes[^32^, ^33^] suggest (a) that such fragments dissociate heterolytically over metal oxide surfaces under anaerobic conditions, and (b) that the Lewis acid‐base interaction energies of dissociated R^−^ and H^+^ fragments on metal oxide surfaces are surface‐ and molecule‐dependent and must be considered in addition to the DPEs. For example, heterolytic R−H scission has previously been invoked to explain anaerobic SHC over Bi_2_O_3_ catalysts,^[9]^ and DFT computations show that Bi−O site pairs facilitate favorable hydride binding energies relative to other R^−^ groups (e.g., CH_3_^−^, C_2_H_3_^−^, C_3_H_5_^−^). For Na_2_WO_4_/SiO_2_, given that molecular descriptors alone fail to explain high hydrogen combustion selectivity during aerobic SHC, we posit that the surface is able to generate specific oxygen species (e.g., O_2_^2−^, as reported in the OCM literature)[^11^, ^19^, ^34^] which preferentially interact with H−H pairs compared with C_m_H_n−1_−H pairs. Below, we discuss the chemical and structural characteristics of the catalyst which facilitate these preferential interactions.

Na and W are both essential components of the catalyst formulation, as neither NaO_x_/SiO_2_ (nominal Na loading of 0.8 wt %) nor unsupported WO_3_ gives the necessary combination of high rate and high (>90 %) combustion selectivity necessary for SHC at 923 K. NaO_x_/SiO_2_ has previously been shown to have a very high (300 kJ mol^−1^) barrier for O_2_ activation^[35]^ and gives very low hydrogen combustion rates (Figure S2) compared with Na_2_WO_4_/SiO_2_ (Figure 1a); for identical Na loadings, combustion STYs in CH_4_−H_2_−O_2_ mixtures (5 kPa CH_4_, 5 kPa H_2_, 1.25 kPa O_2_) are ca. 70× lower at 923 K and 120× lower at 983 K for NaO_x_/SiO_2_ (Figure S2) than for Na_2_WO_4_/SiO_2_ (Figure 1). By contrast, while WO_3_ selectively combusts hydrogen in H_2_−CH_4_−O_2_ (4 : 4 : 1) mixtures with a comparable apparent activation energy (104±4 kJ mol^−1^, Figure S3b) to Na_2_WO_4_/SiO_2_, (115±5 kJ mol^−1^, Figure S3a), the CO_x_ formation rate in the corresponding H_2_−C_3_H_6_−O_2_ (4 : 4 : 1) experiment is similar to the H_2_O formation rate at 903 K (Table S9). Thus, pure WO_3_ is a relatively unselective catalyst for hydrogen combustion in the presence of propylene. We surmise that the alkali metal component is necessary to attenuate alkene combustion rates while also promoting hydrogen combustion. An additional OCM‐active alkali metal‐promoted catalyst, Li/MgO, was also demonstrated to be selective for SHC compared with unpromoted MgO (Figure S4), supporting the postulate that hydrogen‐selective species form in the presence of alkali metal sites.

Alkali cations have been previously shown to be responsible for activation of H_2_O in the presence of O_2_ during OCM over alkali tungstate and molybdate materials.^[12]^ The prior literature in OCM catalysis over Na_2_WO_4_ has debated the identity of the oxygen species involved in C−H activation; recent ambient pressure X‐ray photoelectron spectroscopy (AP‐XPS) measurements of alkali metal‐based catalysts[^11^, ^19^, ^36^] demonstrate that reactive peroxide and superoxide intermediates form over alkali metal cations in O_2_ environments. For K_2_WO_4_/SiO_2_ catalysts, these features appear in the temperature range 833–953 K,^[19]^ similar to the conditions in our study; thus, dioxygen species which form over alkali cations may also be relevant for SHC over Na_2_WO_4_/SiO_2_. Noting that calculated H adsorption energies (HAEs) on O_2_^2−^ species have been reported to be 217 kJ mol^−1^ lower than on lattice O^2−^ for La_2_O_3_ OCM catalysts,^[37]^ and that alkali metal peroxides are known to activate CH_4_ anaerobically,[^38^, ^39^] we posit that peroxides may serve as active oxygen species for H−H activation during SHC catalysis by Na_2_WO_4_/SiO_2_.

As a postulate for why such species could be H_2_ selective, we note that the C−O bond dissociation energy in methyl hydroperoxide (279 kJ mol^−1^) is significantly lower than the H−O BDE in hydrogen peroxide (369 kJ mol^−1^), and that formation of OO−H bonds is enthalpically favorable relative to OO−CH_3_ bonds. Proximal surface peroxides can form upon dissociation of dioxygen on metal oxide surfaces (2 O^2−^_(s)_+O_2(g)_→2 O_2_^2−^_(s)_).^[37]^ While radical mechanisms are proposed to dominate in OCM, suggesting that the entropic favorability of forming unbound CH_3_⋅ at high temperature outweighs the enthalpic favorability of forming an OO−C bond, the BDEs listed above suggest that formation of an OO−H bond is significantly more favorable enthalpically, such that both H atoms in a hydrogen molecule could bind to proximal peroxide species to form two surface‐bound OO−H groups, as depicted in Scheme 2, rather than forming one surface‐bound OO−H and one gaseous H⋅. Such a step would be expected, according to Brønsted‐Evans‐Polanyi relations, to have a significantly lower activation barrier than the homolytic step suggested for C−H activation, such that hydrogen is able to scavenge active surface peroxide species more rapidly than other hydrocarbons.

**Scheme 2:**
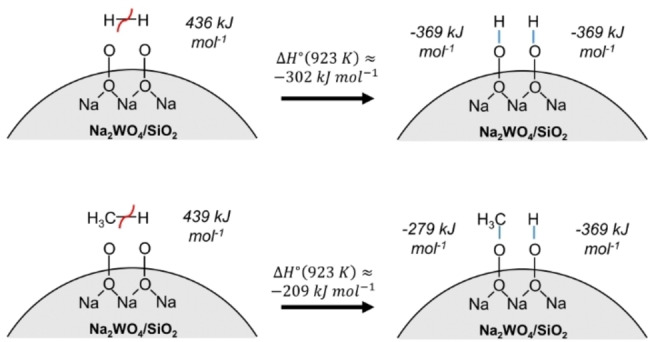
Schematic of direct H−H or H_3_C−H dissociation over proximal surface peroxides, suggested to exist from DFT^[37]^ and AP‐XPS[^11^, ^19^] results. Red curved lines depict bonds broken in the reactant state, while blue lines depict bonds formed in the product state; bond enthalpies in the product state are estimated from R−OO BDEs of hydrogen peroxide (for H−OO) and methyl hydroperoxide (for H_3_C−OO). The reaction enthalpy is ca. 93 kJ mol^−1^ more favorable for formation of an OH−OH site pair than an OCH_3_−OH site pair. WO_x_ groups are not depicted but are expected to be proximal to Na^+^, as suggested by Wachs and co‐workers.^[40]^

In addition to promoting active oxygen species formation, Na^+^ cations are known to act as chemical promoters which remove the surface acidity of WO_x_, as quantified by NH_3_‐TPD experiments comparing WO_x_/SiO_2_ and Na−WO_x_/SiO_2_ materials.^[16]^ Acidic sites would otherwise be expected to interact with alkene π bonds, enabling facile combustion of C_3_H_6_ or other alkenes in the absence of Na^+^. Together, these arguments account for the activity and selectivity enhancement induced by Na^+^.

While Na_2_WO_4_/SiO_2_ contains crystalline SiO_2_ and Na_2_WO_4_ phases under ambient conditions both before and after reaction (Figure S7), high‐temperature XRD measurements demonstrate that the Na_2_WO_4_ phase lacks long‐range order under SHC reaction conditions (923 K, ca. 1 bar total pressure), even though the temperature of the catalyst is below the bulk melting point of pure Na_2_WO_4_ at atmospheric pressure (971 K). In situ XRD measurements were done in flowing N_2_ up to 983 K and diffractograms from these measurements are shown in Figure S8. In addition to a cristobalite SiO_2_ phase which transforms from the α to β polymorph at 298 K<T<773 K,^[13]^ peaks corresponding to cubic Na_2_WO_4_ were observed in XRD measurements taken in air up to T≤948 K. The intensity of these peaks began to decrease in the range 873 K≤T≤948 K to a greater extent than would be expected from thermal excitations of the crystalline lattice alone. This suggests a partial loss of long‐range order below the melting temperature, before complete melting at T≤973 K. Na_2_WO_4_ did not recrystallize upon cooling to 923 K, consistent with prior observations by Werny et al. for Mn/Na_2_WO_4_/SiO_2_ catalysts.^[41]^ The standard pretreatment procedure for the reaction experiments involves heating to 983 K before cooling to 923 K for SHC, and Na_2_WO_4_ is therefore expected to be either a melt or an amorphous solid during SHC reactions.

W L_III_‐edge XAS spectra demonstrate that there is a loss in second‐shell W coordination even at temperatures well below the melting point, such that long‐range order with respect to W is lost well before melting. Figure 4 and Figure S9 show normalized XANES, |χ(R)|, and χ(k) EXAFS spectra of Na_2_WO_4_/SiO_2_ obtained in helium in the range 293 K≤T≤983 K. The edge energies determined from the XANES spectra are identical (within ±0.2 eV) at all temperatures to the edge energy of the solid Na_2_WO_4_ ⋅ 2 H_2_O reference material, consistent with a W^6+^ oxidation state at all conditions. Figure 4b demonstrates a decrease in second coordination shell features with increasing temperature well below the bulk Na_2_WO_4_ melting temperature, and complete disappearance of any second coordination shell features at 873 K. Second‐shell features were not observed upon rapid cooling of the sample from 873 K to ambient temperature (Figure S11) and the 873 K and quenched sample χ(R) spectra are nearly identical, evincing that the loss of second‐shell features reflects a structural change rather than increasing thermal disorder alone. These data suggest destructive interference of individual scatterer contributions to χ(k) in the range of the second coordination shell, and thus show a departure from the behavior expected for a crystalline sample, in which there is long‐range order with respect to all W atoms. EXAFS fits for average W coordination number, shown in Table S10 and Figure S10, are ca. 3.5±0.5 at all temperatures. Significant shifts in the position of the first‐shell peak in |χ(R)| are not observed and W−O interatomic distances are 1.78±0.01 Å at all temperatures, similar to W−O bond lengths in other tetrahedrally coordinated compounds^[42]^ and consistent with the 1.783 Å W−O spacing of cubic Na_2_WO_4_ measured at ambient temperature.^[43]^

**Figure 4:**
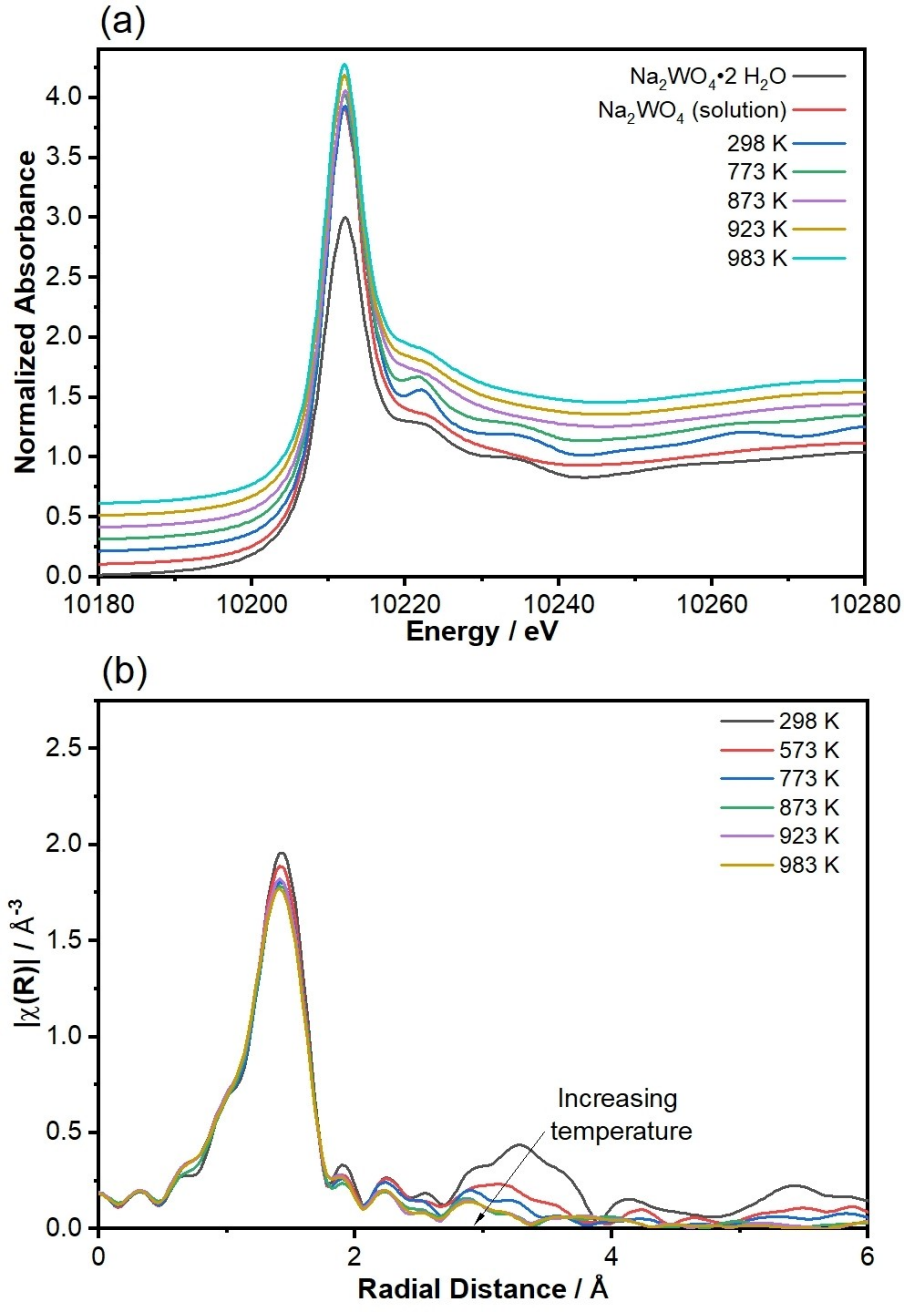
Comparison of W L_III_‐edge (a) XANES and (b) k^2^‐weighted |χ(R)| spectra for Na_2_WO_4_/SiO_2_ samples held in He at varying temperatures. XANES spectra of Na_2_WO_4_ standards are given for comparison; frozen Na_2_WO_4_ solution spectra were obtained from Ref. [46]. Normalized μ(E) are offset in increments of 0.1. EXAFS spectra at 873 K, 923 K, and 983 K are nearly identical and are indistinguishable in the |χ(R)| plot.

The XANES spectra shown in Figure 4a demonstrate that the catalyst at T≥873 K has characteristics similar to solvent‐separated Na^+^ and WO_4_^2‐^,[^44^, ^45^, ^46^] rather than crystalline Na_2_WO_4_ ⋅ 2 H_2_O. Given that XAS measures contributions of all W atoms while XRD measures crystalline contributions only, the XANES and second‐shell EXAFS results in Figure 4 and Figure S11 suggest that a significant fraction of W atoms are not present in the cubic Na_2_WO_4_ lattice even though a bulk crystalline phase exists prior to melting according to XRD. Such sites could instead be dispersed on the support surface as non‐crystalline (Na)−WO_x_ sites, as Kiani et al.[^16^, ^40^] concluded according to in situ Raman measurements. Plausibly, alkali metal sites proximal to such WO_x_ groups facilitate the formation of the oxygen species active and selective for SHC.

Collectively, these results demonstrate that the Na_2_WO_4_/SiO_2_ formulation studied herein forms a stable, disordered, and highly selective catalyst for aerobic hydrogen combustion at elevated temperatures (883–983 K), and is representative of a new class of alkali metal‐based SHC catalysts. The remarkable selectivity (>97 %) for hydrogen combustion in equimolar mixtures with hydrocarbons is observed for hydrocarbons (e.g., C_3_H_6_) with both weaker bond energies and higher acidities compared with H_2_. Given the effectiveness of Na_2_WO_4_/SiO_2_ catalysts for SHC over a wide range of operating conditions, including for mixtures in which hydrocarbons are fed in significant excess, we posit that DH+aerobic SHC processes involving Na_2_WO_4_/SiO_2_ could enable significant olefin yield enhancements at high temperatures that facilitate more rapid olefin formation.

## Supporting Information

The authors have cited additional references within the Supporting Information.[^24^, ^44^, ^45^, ^46^, ^47^, ^48^, ^49^, ^50^, ^51^, ^52^]

## Conflict of Interests

The authors declare no conflict of interest.

## Supporting information

As a service to our authors and readers, this journal provides supporting information supplied by the authors. Such materials are peer reviewed and may be re‐organized for online delivery, but are not copy‐edited or typeset. Technical support issues arising from supporting information (other than missing files) should be addressed to the authors.

Supporting Information

## Data Availability

The data that support the findings of this study are available from the corresponding author upon reasonable request.
