# Supplementary material for: Selective Catalytic Combustion of Hydrogen under Aerobic Conditions on Na2WO4/SiO2
Source: Angew Chem Int Ed Engl. 2024 Oct 29;63(52):e202412932. doi: 10.1002/anie.202412932 (PMC11656149; doi:10.1002/anie.202412932)
Supplement: Supplementary file 1 — Supporting Information [file ANIE-63-e202412932-s001.pdf]

Angewandte Chemie
Eine Zeitschrift der Gesellschaft Deutscher Chemiker

Supporting Information

Selective Catalytic Combustion of Hydrogen under Aerobic Conditions on Na_2WO_4/SiO_2

*E. R. Kipp, J. Garcia-Barriocanal, A. Bhan**

Angewandte Chemie
Eine Zeitschrift der Gesellschaft Deutscher Chemiker

Supporting Information

Selective Catalytic Combustion of Hydrogen under Aerobic Conditions on Na_2WO_4/SiO_2

*E. Kipp, J. Garcia-Barriocanal, A. Bhan**

Supporting Information

Selective Catalytic Combustion of Hydrogen under Aerobic Conditions on Na_2WO_4/SiO_2

E. Kipp, J. Garcia-Barriocanal, A. Bhan*

[*] Department of Chemical Engineering and Materials Science
University of Minnesota, Twin Cities
421 Washington Ave. SE, Minneapolis, Minnesota 55455, USA
E-mail: abhan@umn.edu

Table of Contents

S1. Experimental Section ... 3
S1.1. Synthesis of Na_2WO_4/SiO_2, Na/SiO_2, Li/MgO, and sand diluent ... 3
S1.2. Catalytic reaction experiments ... 3
S1.3. X-ray absorption spectroscopy (XAS) ... 5
S1.4. X-ray diffraction (XRD) ... 6
S2. Calculations for absence of transport limitations ... 7
S2.1. Mears's criterion: interphase mass transfer limitations ... 7
S2.2. Mears's criterion: interphase heat transfer limitations ... 9
S2.3. Effectiveness factor: intraparticle mass transfer limitations ... 10
S2.4. Mears's criterion: intraparticle heat transfer limitations ... 11
S2.5. Mears's criterion: radial temperature gradients ... 12
S3. Experimental absence of transport limitations, bed-scale gradients, and non-catalytic reactions ... 13
S3.1. Empty and blank bed experiments ... 13
S3.2. Particle size tests ... 14
S4. Calculation of conversion, selectivity, and rate ... 16
S5. SHC experiments over NaO_x/SiO_2 and WO_3 ... 17
S5.1. NaO_x/SiO_2 SHC experiments ... 17
S5.2. WO_3 SHC experiments ... 17
S5.3. Li/MgO SHC Experiments ... 19
S6. Kinetic and isotopic experiments over Na_2WO_4/SiO_2 ... 20
S6.1. H_2 and C_2H_4 combustion selectivity over changes in O_2 pressure ... 20
S6.2. Invariance of H_2 combustion rates with contact time ... 20
S6.3. $^{16}O_2$-$^{18}O_2$ isotopic scrambling experiments ... 21
S7. Characterization of Na_2WO_4/SiO_2 ... 23
S7.1. Ex situ XRD for pre- and post-reaction samples ... 23
S7.2. High temperature XRD patterns for Na_2WO_4/SiO_2 in air ... 23
S7.3. X-ray absorption spectroscopy at SHC temperatures ... 25
References ... 29

S1. Experimental Section

S1.1. Synthesis of Na_2WO_4/SiO_2, Na/SiO_2, Li/MgO, and sand diluent

5 wt. % Na_2WO_4/SiO_2 was synthesized by incipient wetness impregnation following a procedure adapted from Kiani et al.[1,2] Fumed SiO_2 (CAB-O-SIL M 5, Cabot Corporation) was first treated with excess water (HPLC grade, Fisher Scientific, ~6 g water per g SiO_2), held overnight at ambient temperature, and then dried in stagnant air at 393 K for 8 h. The SiO_2 was subsequently treated in dry flowing air (3.33 cm^3 (STP) s^{-1}) at 773 K for 4 h following a 0.083 K s^{-1} temperature ramp. The SiO_2 obtained after the water treatment step was sieved to form a powder comprising 177-425 μm particles.

0.665 g of $Na_2WO_4 \bullet 2\ H_2O$ (>99.0%, Sigma Aldrich) was dissolved in 10.035 g of HPLC water at ambient temperature to form a 6.2 wt % Na_2WO_4 aqueous solution. 0.91 g of solution was added dropwise to the water-treated SiO_2 support. After impregnation, the resulting materials were dried overnight in ambient air, further dried in stagnant air at 383 K for 1 h, and then treated in dry flowing air (3.33 cm^3 (STP) s^{-1} g_{SiO2}^{-1}) at 1173 K for 8 h following a 0.083 K s^{-1} temperature ramp.

0.8 wt. % NaO_x/SiO_2 was prepared by the same method as 5 wt. % Na_2WO_4/SiO_2, except that an aqueous solution of 1.6 wt. % NaOH (>98.7%, Fisher Scientific) used to impregnate the water-treated support instead of 6.2 wt. % Na_2WO_4.

Li/MgO was prepared by combining 0.1 g of Li_2CO_3 (99.997%, Alfa Aesar) with 2.5 g of MgO (99.99%, Alfa Aesar) and mixing in 60 cm^3 of HPLC water. The resulting slurry was held at 358 K for 8 h until nearly all of the water had evaporated. The recovered solids were then dried completely in a vacuum oven held at 413 K for 8 h. Next, the solids were treated in 1.67 cm^3 (STP) s^{-1} of dry flowing air at 873 K for 12 h following a 0.083 K s^{-1} temperature ramp.

Sand was prepared as a bed diluent; calculations in the next section demonstrate that an inert solid diluent is necessary to avoid radial heat transport limitations in the catalyst bed.[3] 40 g of sand (Acros Organics, 40-100 mesh) was mixed with 200 cm^3 of 2 M nitric acid (Fisher Chemical) in a round-bottom flask and stirred slowly with a glass-lined stir rod for 24 h. The sand was subsequently washed with water (HPLC grade, Fisher Scientific) until the rinse had a neutral pH, dried in stagnant air at 393 K for 6 h, and treated in dry flowing air (3.33 cm^3 (STP) s^{-1}) at 1273 K for 16 h following a 0.167 K s^{-1} temperature ramp. The sand was not crushed or sieved prior to catalytic testing.

S1.2. Catalytic reaction experiments

Fused quartz rods (Technical Glass Products) and quartz wool (CE Elantech) were held overnight in 0.6 M nitric acid (Fisher Chemical), rinsed to a neutral pH, and dried prior to loading reactor tubes.

Na_2WO_4/SiO_2 powder was pressed (~3500 psi), crushed, and sieved to retain 177-425 μm particles. 20-100 mg of sieved powder was then physically mixed with ~250 mg of acid-washed sand and

loaded into a vertically mounted 10.5 mm ID × 12.75 mm OD fused quartz reactor tube (Technical Glass Products). Other catalyst powders (NaO_x/SiO_2, WO_3, Li/MgO) were sieved to the same particle size; NaO_x/SiO_2 was not diluted, while WO_3 (39 mg) and Li/MgO (25 mg) were diluted with ~250 mg sand. The catalyst powder was supported by plugs of acid-washed quartz wool above and below the catalyst bed. 10 mm diameter acid-washed fused quartz rods were placed above and below the catalyst bed and extended beyond the heated zone of the reactor tube; significant CH_4 and H_2 combustion occurred in an empty reactor tube at 983 K when the glass rods were not present. The reactor tube was placed in an annular metal block held within an electric furnace (National Element, FA-120). A thermocouple on the outside of the metal block provided the signal to a PID controller (Watlow, EZ Zone) which regulated power to the furnace.

Mass flow controllers (Brooks Instrument) were used to control the flow of hydrogen (99.9999%, Airgas), methane (99.999%, Airgas), ethane (99.995%, Matheson Tri-Gas), ethylene (99.95%, Matheson Tri-Gas), propylene (99.99%, Matheson Tri-Gas), deuterium (99.995%, Matheson Tri-Gas), air (20.9% O_2, balance N_2, <8 ppm impurities, Matheson Tri-Gas), and helium (99.9995%, Airgas). To flow benzene (>99.9%, Sigma Aldrich) to the reactor, liquid was introduced via a syringe pump (Cole-Palmer 78-0100C) downstream of the gas manifold through a stainless-steel T-junction (Swagelok) packed with quartz wool (CE Elantech). The T-junction was held at ~10 K below the boiling point of the liquid so that the liquid could evaporate into ≥1.67 cm^3 (STP) s^{-1} cross-flowing gas.

The catalyst was pretreated in 0.25 cm^3 (STP) s^{-1} helium during a 0.083 K s^{-1} heating step from ambient temperature to 983 K. The catalyst was then held for 14 h in a 1.67 cm^3 (STP) s^{-1} mixture containing 5 kPa CH_4, 5 kPa H_2, 1.25 kPa O_2, balance He. The reactor was subsequently cooled to the reaction temperature (873-973 K) at -0.083 K s^{-1} in the same reactant gas mixture for reaction experiments. On-line quantification of reactants, products, and inert gases (except He) was performed by gas chromatography (GC) (Agilent 7890A), using a thermal conductivity detector (TCD) and flame ionization detector (FID) to measure gas composition. An HP-PLOT Q column was used to facilitate the separation of H_2, N_2, O_2, CO, CH_4, CO_2, C_2H_6, C_2H_4, C_3H_6, H_2O, and C_6H_6. Helium (99.9995%, Airgas) was used as the carrier gas and as the reference gas in the thermal conductivity detector. Hydrocarbon conversions did not exceed 0.2% under any conditions (e.g., $X_{CH4} \sim 0$ and $X_{C2H4} = 0.0006$ in Fig. 1b), the cofed hydrocarbon was used as an internal standard for each experiment. Liquid nitrogen was used as a cryogen to separate H_2, N_2, O_2, CO, and CH_4 peaks during chromatographic analysis. The GC oven was held at 213 K for 5 min. before ramping at 40 K min^{-1} to 513 K and holding for 2.5 min. A sample TCD chromatogram is shown in Fig. S1.

For $^{16}O_2/^{18}O_2$ isotopic experiments, 5% O_2/Ar (Airgas, Certified) was used as the $^{16}O_2$ source. A 5.0% $^{18}O_2$/He cylinder was prepared by first vacuum purging a 150 cm^3 sample cylinder (Swagelok) with a rotary vane vacuum pump connected to the cylinder outlet (Pfeiffer Duo 2.5). A shutoff valve was used to close the cylinder at the outlet before filling it with other gases. At the cylinder inlet, a three-way valve was used to switch between He (99.9995%, Airgas) and $^{18}O_2$

(Cambridge Isotope Laboratories, 97% $^{18}O_2$, 99.8% chemical purity) gases. The cylinder was first pressurized to 35 kPa with $^{18}O_2$, and subsequently filled with He to a final pressure of 698 kPa.

Pretreatment procedures for $^{16}O_2$/$^{18}O_2$ experiments were identical to the pretreatment procedures other catalytic experiments; $^{18}O_2$ was not used during pretreatment. An online mass spectrometer (MKS Cirrus 200 quadrupole MS system) downstream of the reactor bed was used to detect m/z = 18 (H_2O), 32 ($^{16}O_2$), 34 ($^{16}O^{18}O$), 36 ($^{18}O_2$), and 40 (Ar) fragments. Ar was used as an internal standard.

S1.3. X-ray absorption spectroscopy (XAS)

W L_{III}-edge XAS measurements were made at beamline 9-3 at the Stanford Synchrotron Radiation Lightsource (SSRL). A liquid nitrogen-cooled, double-crystal Si(220) monochromator was used to select photon energies. Spectra were collected in transmission mode using N_2-filled ion chambers to monitor the relative intensity of the X-ray beam before and after the sample, and before and after the reference foil. A W foil located downbeam of the sample was used as an internal reference to calibrate the photon energy of each spectrum. Continuous extended X-ray absorption fine structure (EXAFS) spectra were measured from 200 eV below the edge (10207 eV) to a wavenumber of k = 16 $Å^{-1}$ (11182 eV) with a 90 s scan time.

To measure spectra for Na_2WO_4●$2H_2O$ powder references, materials were mixed with cellulose in a (Na_2WO_4●$2H_2O$:cellulose weight ratio of 10:1), pressed into a 7 mm diameter pellet, and loaded onto Kapton tape to enable measurement of the W L_{III}-edge at ambient conditions. The 0.1 M Na_2WO_4 solution reference spectrum was collected by Proux and coworkers[4,5] and obtained from the SSHADE database.[6]

To measure spectra for Na_2WO_4/SiO_2, approximately 30 mg of sample was loaded into a quartz capillary (O.D. = 3 mm) positioned perpendicular to the beam to enable *in situ* measurement of the W L_{III}-edge. XAS measurements were obtained from 298-983 K in the presence of 0.33 cm^3 (STP) s^{-1} of flowing He. The temperature was increased at 0.083 K s^{-1} and data were collected continuously during the temperature ramp. At several temperatures (298 K, 773 K, 873 K, 923 K, 983 K), the temperature was held constant so that four scans could be collected and averaged to improve the signal-to-noise ratio at these conditions.

Athena and Artemis software[7] were used in the analysis of XAS data. Pre-processing of data was performed in Athena and included alignment, edge calibration, background subtraction, and normalization. Spectra were aligned by setting the first maximum of the first derivative of the absorption edge of the tungsten foil reference to the reported W L_{III}-edge energy of 10207.0 eV.

The amplitude reduction factor of the beamline (S_0^2 = 0.95) was obtained by fitting the EXAFS region of the W foil standard to its crystal structure. EXAFS fits were performed with k-weights of 1, 2, and 3 in the range 3.8 $Å^{-1} \leq k \leq 13.7$ $Å^{-1}$ with 1.0 Å $\leq r \leq$ 2.1 Å and a Hanning window with dk = 1.0 Å. CIF files for EXAFS fitting were obtained from the Materials Project database.

S1.4. X-ray diffraction (XRD)

XRD patterns were obtained with a Rigaku SmartLab XE diffractometer using Cu-Kα radiation (λ = 0.154 nm) in Bragg-Brentano configuration. For measurements at ambient conditions, diffraction patterns were recorded from 2θ in the range 5-80°, with the detector moving in Δ2θ = 0.005° steps at a rate of 4° min^{-1}. For non-ambient measurements, the sample was held in 0.083 cm^3 (STP) s^{-1} of flowing N_2, diffraction patterns were recorded from 2θ in the range 15-40°, with the detector moving in Δ2θ = 0.005° steps at a rate of 1.2° min^{-1}. Heating was realized using a DHS 1100 domed hot stage (Anton Paar), and the sample was heated at 0.167 K s^{-1} between acquisition of successive XRD patterns in N_2.

Simulated diffraction patterns for cubic Na_2WO_4 (space group: Fd-3m) and α-cristobalite (space group: $P4_12_12$) were obtained by using crystallographic information from the Materials Project database and calculating patterns using VESTA software.

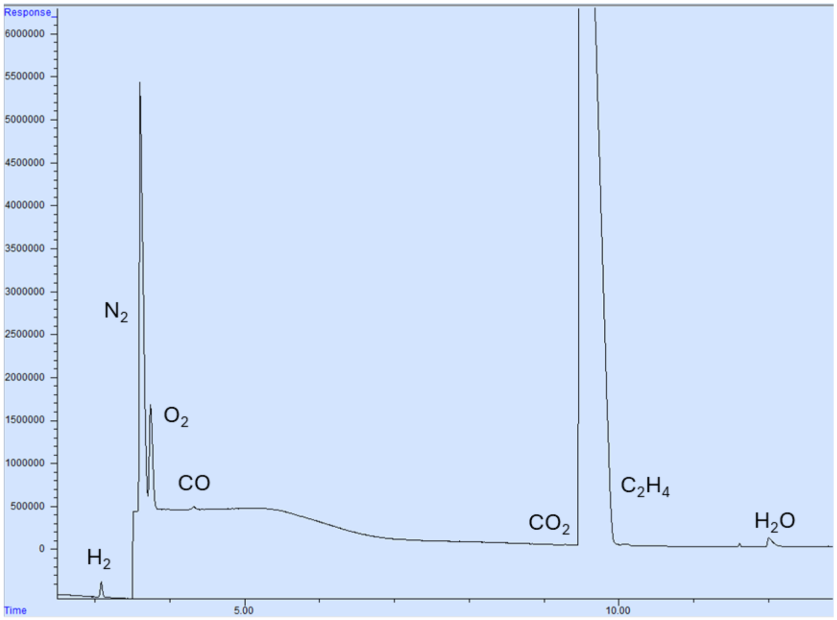

Figure S1: *Sample TCD chromatogram for a reactor influent of 40 kPa C_2H_4, 5 kPa H_2, 4.7 kPa N_2, and 1.25 kPa O_2. Positions of reactant and product peaks are labeled on the chromatogram.*

S2. Calculations for absence of transport limitations

The sample calculations are given for a reference catalytic condition: 5 kPa CH_4, 5 kPa H_2, 1.25 kPa O_2, balance He, 1.67 cm^3 (STP) s^{-1} total flow rate, 923 K, 1.17 bar total pressure. Reactor tube: 10.5 mm ID. Catalysts were sieved to retain 177-425 μm aggregates; an average particle diameter of 300 μm is assumed in all cases.

S2.1. Mears's criterion: interphase mass transfer limitations

Variables are defined and data for the external mass transfer calculation are given in Table S1.

Table S1 *Reaction conditions, measured parameters, physical property data, and calculated parameters for Mears's interphase mass transfer criterion. Binary diffusion coefficients were calculated according to the method outlined by Fuller et al.*[8] *Helium dynamic viscosities calculated using a correlation from Petersen.*[9]

Parameter	Variable	Parameter Value
Average particle radius	r_P	1.5×10^{-4} m
Gas-phase H_2 concentration (923 K, 1.25 kPa O_2)	$C_{A,b}$	0.65 mol m^{-3}
Catalyst powder bulk density	ρ_b	500 kg m^{-3}
H_2 reaction order	n	0.8
Observed H_2 combustion rate	$-r_{A,obs}$	~5 x 10^{-3} mol kg_{cat}^{-1} s^{-1}
Bed void fraction [assumed]	ϕ_b	0.5
Dynamic viscosity of gas mixture	μ	4.4×10^{-5} Pa s
Superficial gas velocity (He, 923 K, 1.17 bar)	U_s	0.0513 m s^{-1}
Binary diffusion coefficient of H_2 in He	D_{AB}	1.08×10^{-3} m^2 s^{-1}
Catalyst pellet density	ρ_s	1000 kg m^{-3}
Schmidt number	Sc	0.71
Reynolds number	Re	0.023
Sherwood number	Sh	2.08
Mass transfer coefficient	k_c	7.45 m s^{-1}
Mears's criterion for external mass transfer	-	1.3×10^{-4}

Mears showed[3] that external mass transfer limitations can be neglected when:

$$-\frac{r_{A,obs}\rho_s r_P n}{k_c C_{A,b}} < 0.15 \qquad \text{(S2.1)}$$

Where the pellet solid density is calculated according to:

$$\rho_s = \frac{\rho_b}{1-\phi_b} \qquad \text{(S2.2)}$$

Species A is chosen to be H_2 here, as the H_2 reaction order is much larger (0.8) than that for O_2 (<0.1) and as such O_2 concentration gradients are therefore unlikely to be kinetically-relevant.

k_C is calculated from the Sherwood number Sh and is related to the Reynolds number Re and Schmidt number Sc according to Eqs. S2.3-S2.5:

$$Sh = \frac{2k_c r_p}{D_{AB}} = 2 + 0.6Re^{0.5}Sc^{0.33} \tag{S2.3}$$

$$Re = \frac{2\rho_g r_P U_s}{\mu} \tag{S2.4}$$

$$Sc = \frac{\mu}{\rho_g D_{AB}} \tag{S2.5}$$

The calculated value of Mears's criterion using the data provided in Table S1 is 1.3 x 10^{-4}, confirming the absence of significant mass transfer limitations.

S2.2. Mears's criterion: interphase heat transfer limitations

Variables are defined and data for the external heat transfer calculation are given in Table S2.

Table S2 *Reaction conditions, measured parameters, physical property data, and calculated parameters for Mears's interphase heat transfer criterion. Helium thermal conductivity was calculated using a correlation from Petersen.*[9]

Parameter	Variable	Parameter Value
Reaction enthalpy (H_2 + ½ O_2 → H_2O)	ΔH_{rxn}	2.46×10^5 J mol^{-1}
Apparent activation energy	E_{app}	1.1×10^5 J mol^{-1}
Catalyst powder bulk density	ρ_b	500 kg m^{-3}
Average particle radius	r_P	1.5×10^{-4} m
Observed H_2 combustion rate	$-r_{A,obs}$	~5×10^{-3} mol kg$_{cat}^{-1}$ s^{-1}
Bed void fraction [assumed]	ϕ_b	0.5
Bed (set) temperature	T_b	923 K
He thermal conductivity	λ	0.34 W m^{-1} K^{-1}
Gas constant	R_g	8.314 J mol^{-1} K^{-1}
Catalyst pellet density	ρ_s	1000 kg m^{-3}
Reynolds number	Re	0.023
Nusselt number	Nu	2 [Assumed for low Re]
Heat transfer coefficient	h	2.23×10^3 W m^{-2} K^{-1}
Mears's criterion for external heat transfer	-	3.8×10^{-5}

Mears showed[3] that external heat transfer limitations can be neglected when:

$$\left|\frac{-\Delta H_{rxn}(-r_{A,obs})\rho_s r_P E_{app}}{h T_b^2 R_g}\right| < 0.15 \quad \text{(S2.6)}$$

where h is calculated from the Nusselt number Nu, assumed to be 2 at low Re.

$$Nu = \frac{2 r_P h}{\lambda} \approx 2 \quad \text{(S2.7)}$$

The apparent activation energy of H_2 is calculated from the Arrhenius plot in Fig. S1.

The calculated value of Mears's criterion using the data provided in Table S2 is 3.8×10^{-5}, confirming the absence of significant external heat transfer limitations.

S2.3. Effectiveness factor: intraparticle mass transfer limitations

Variables are defined and data for the intraparticle mass transfer calculations (effectiveness factor calculations) are given in Table S3.

Table S3*: Reaction conditions, measured parameters, physical property data, and calculated parameters for the catalyst effectiveness factor during SHC. Typical values*[10] *of bed void fraction, pellet void fraction, and pellet pore tortuosity are assumed. Binary diffusion coefficients were calculated according to the method outlined by Fuller et al.*[8]

**Parameter**	**Variable**	**Parameter Value**
Average particle radius	r_P	1.5×10^{-4} m
Surface H_2 concentration [assume equal to $C_{A,b}$ since there are no external mass transfer gradients]	$C_{A,s}$	0.65 mol m^{-3}
Catalyst powder bulk density	ρ_b	500 kg m^{-3}
H_2 reaction order	n	0.8
Observed H_2 combustion rate	$-r_{A,obs}$	~5×10^{-3} mol kg_{cat}^{-1} s^{-1}
Bed void fraction [assumed]	ϕ_b	0.5
Pellet void fraction [assumed]	ϕ_P	0.35
Pellet pore tortuosity [assumed]	τ	7
Binary diffusion coefficient of H_2 in He	D_{AB}	1.08×10^{-3} m^2 s^{-1}
Effective diffusivity	D_{eff}	5.4×10^{-5} m^2 s^{-1}
Catalyst pellet density	ρ_S	1000 kg m^{-3}
Thiele modulus	Φ	0.0179
Effectiveness factor	η	0.9999

The Thiele modulus can be calculated from the following equation:

$$\Phi = \left[\left(\frac{R}{3}\right)^2\left(\frac{n+1}{2}\right)\left(\frac{-r_{A,obs}\rho_s}{D_{eff}C_{A,s}}\right)\right]^{1/2} \quad \text{(S2.8)}$$

where the effective diffusivity is:

$$D_{eff} = \frac{D_{AB}\phi_p}{\tau} \quad \text{(S2.9)}$$

The effectiveness factor can be estimated from the Thiele modulus:

$$\eta \approx \frac{\tanh\Phi}{\Phi} \quad \text{(S2.10)}$$

The calculated effectiveness factor of 0.9999 is very close to the ideal case with no internal mass transfer limitations ($\eta = 1$); thus, internal mass transfer limitations are not present.

S2.4. Mears's criterion: intraparticle heat transfer limitations

Variables are defined and data for the intrapellet heat transfer calculation are given in Table S4.

Table S4: *Reaction conditions, measured parameters, physical property data, and calculated parameters for Mears's intraparticle heat transfer criterion. The solid thermal conductivity was estimated from the measured thermal conductivity of β-cristobalite at 923 K.*[11] *The calculation of the pellet density is given in the previous section.*

Parameter	Variable	Parameter Value
Reaction enthalpy ($H_2 + ½ O_2 \rightarrow H_2O$)	ΔH_{rxn}	2.46×10^5 J mol^{-1}
Apparent activation energy	E_{app}	1.1×10^5 J mol^{-1}
Catalyst pellet density	ρ_s	1000 kg m^{-3}
Average particle radius	r_P	1.5×10^{-4} m
Observed H_2 combustion rate	$-r_{A,obs}$	~5×10^{-3} mol kg$_{cat}^{-1}$ s^{-1}
Surface temperature [assume equal to T_b since there are no external thermal gradients]	T_s	923 K
Solid thermal conductivity	λ_s	1.5 W m^{-1} K^{-1}
Pellet void fraction [assumed]	ϕ_P	0.35
Gas constant	R_g	8.314 J mol^{-1} K^{-1}
Pellet thermal conductivity	λ_P	0.98 W m^{-1} K^{-1}
Mears's criterion for isothermal pellets	-	4.4×10^{-4}

Mears showed[3] that intrapellet thermal gradients can be neglected when:

$$\frac{-r_{A,obs}\rho_s|\Delta H_{rxn}|Er_P^2}{\lambda_p R_g T_s^2} < 1 \quad \text{(S2.11)}$$

The thermal conductivity of the pellets is estimated from the solid thermal conductivity of cristobalite and the estimated void fraction of the pellet:

$$\lambda_P \approx \lambda_s(1 - \phi_p) \quad \text{(S2.12)}$$

The calculated value of Mears's criterion for intrapellet thermal gradients using the data provided in Table S4 is 4.4×10^{-4}, confirming the absence of significant external heat transfer limitations.

S2.5. Mears's criterion: radial temperature gradients

Variables are defined and data for the radial heat transfer calculation are given in Table S5.

***Table S5**: Reaction conditions, measured parameters, physical property data, and calculated parameters for Mears's radial thermal gradient criterion. Helium thermal conductivity was calculated using a correlation from Petersen and used as a lower bound for the thermal conductivity of the catalyst bed.[9] Mears[3] states that typical values of the wall Biot number (Bi_W) range from 0.8-2, so 1 was used as an intermediate value here. The diluent : catalyst volume ratio of 3 assumes a bed loading of 0.03 g Na_2WO_4/SiO_2 and 0.25 g sand.*

Parameter	Variable	Parameter Value
Reaction enthalpy ($H_2 + ½ O_2 \rightarrow H_2O$)	ΔH_{rxn}	2.46×10^5 J mol^{-1}
Apparent activation energy	E_{app}	1.1×10^5 J mol^{-1}
Pellet [solid] density	ρ_s	1000 kg m^{-3}
Average particle radius	r_P	1.5×10^{-4} m
Observed H_2 combustion rate	$-r_{A,obs}$	~5×10^{-3} mol kg$_{cat}^{-1}$ s^{-1}
Wall temperature [set by furnace]	T_w	923 K
Wall Biot number [assumed]	Bi_w	1
Bed void fraction [assumed]	ϕ_b	0.5
Effective bed thermal conductivity [set to the thermal conductivity of helium as a lower bound]	λ_b	0.3 W m^{-1} K^{-1}
Diluent : catalyst volume ratio	b	~3
Catalyst bed radius	R_0	5.25×10^{-3} m
Gas constant	R_g	8.314 J mol^{-1} K^{-1}
Reaction rate per bed volume	R_b	0.63 mol m^{-3} s^{-1}
Radial thermal gradients: LHS	-	0.015
Radial thermal gradients: RHS	-	0.025

Mears showed[3] that radial heat transfer gradients can be neglected when:

$$\frac{|\Delta H| R_b R_0^2}{\lambda_b T_W} < \frac{0.4 R_g T_W / E_{app}}{1 + 8 Bi_w^{-1} (r_p / R_0)} \quad \text{(S2.13)}$$

The rate per bed volume is calculated according to:

$$R_b = \frac{1 - \phi_b}{1 + b} \left(-r_{A,obs} \rho_s \right) \quad \text{(S2.14)}$$

The calculated left-hand side of the inequality in Eq. S2.13 is 0.015, while the calculated right-hand side is 0.025; thus, radial thermal gradients can be neglected. We note that when no diluent is used (i.e., b = 0), the criterion in Eq. S2.13 will not be met (assuming the same parameters otherwise) and interparticle radial gradients may be present. An experimental verification for the absence of radial thermal gradients was also performed and is discussed in the following section.

S3. Experimental absence of transport limitations, bed-scale gradients, and non-catalytic reactions

S3.1. Empty and blank bed experiments

Non-catalytic (homogeneous and reactor surface-initiated) combustion routes must be insignificant relative to catalytic routes for kinetic data to be meaningful. Data obtained from catalyst beds loaded only with sand, quartz wool, and quartz filler rods demonstrate that this is the case at 923 or 983 K.

For sand-only experiments, 0.23 g of acid-washed sand was held between 0.05 g total quartz wool; quartz filler rods were also inserted to quench homogeneous reactions. After heating in He to 983 K, the bed was held in a mixture of 5 kPa CH_4, 5 kPa H_2, 1.25 kPa O_2 overnight to simulate the standard pretreatment procedure for Na_2WO_4/SiO_2-containing beds. Results for a typical Na_2WO_4/SiO_2 experiment (bed loading: 0.031 g Na_2WO_4/SiO_2 + 0.25 g acid-washed sand) following the same pretreatment procedure are also given for comparison. H_2O effluent flow rates and calculated O_2 conversions are given in Table S6 for various experimental conditions. CO_x products were not observed in measurable quantities for either the control or catalytic experiments involving CH_4.

Table S6: *Comparison of O_2 conversions and H_2O effluent flow rates in sand-only control experiments versus catalytic experiments. All H_2 and CH_4 partial pressures were set to 5 kPa. Total flow rates in all experiments were 1.67 cm^3 (STP) s^{-1}, with He used as a balance gas. Sand-only experiments: 0.23 g acid-washed sand. Catalytic experiments: 0.031 g catalyst + 0.25 g sand. Large fractional errors are observed in sand-only experiments because measured water peaks are comparable in size to baseline noise.*

Material	Amt. / g	Temp. / K	P_{CH4} / kPa	P_{O2} / kPa	X_{O2}	H_2O Effluent Flow Rate / μmol s^{-1}	Fractional Error (95% CI)
Na_2WO_4/SiO_2	0.031	983	5	1.25	0.21	0.33	±2.7%
Sand	0.23	983	5	1.25	0.012	0.019	±8.4%
Na_2WO_4/SiO_2	0.031	923	5	1.25	0.074	0.11	±8.9%
Sand	0.23	923	5	1.25	0.0032	0.0053	±22%
Sand	0.23	923	5	0.5	0.0042	0.0028	±55%

At 923 K, O_2 conversions in the presence of 0.031 g Na_2WO_4/SiO_2 + 0.25 g sand exceed O_2 conversions in the presence of 0.23 g sand only by a factor of 23×. Thus, measured rates of H_2 combustion primarily reflect reactions initiated on the Na_2WO_4 surface.

In addition, in mixtures of 5 kPa C_3H_6 + 1.25 kPa O_2, the total CO_x formation rate in the presence of sand was 0.0013 μmol s^{-1}. For comparison, the CO_x effluent flow rate from C_3H_6 in the presence of 0.031 g Na_2WO_4/SiO_2 was 0.022 μmol s^{-1}, a 17× difference. C_3H_6 combustion rates are therefore also significantly lower in the absence than in the presence of Na_2WO_4/SiO_2.

S3.2. Particle size tests

The absence of interphase and intraparticle heat and mass transfer gradients during SHC was verified experimentally by validating that the H_2 combustion rate did not change with particle size at a reference condition. One catalyst bed was loaded with particles sieved to the standard size (177-425 μm), while the other was loaded with particles sieved to a smaller size (125-177 μm). Note that the sand diluent in each case was not sieved to the same size as the catalyst particles. Results are shown in Table S7.

Table S7: *H_2O formation rates in catalyst beds containing 0.28 g sand and Na_2WO_4/SiO_2 pellets of different sizes. Reaction conditions: 5 kPa CH_4, 5 kPa H_2, 1.25 kPa O_2, balance He, 1.67 cm^3 (STP) s^{-1}, 923 K. Catalysts were first held overnight at 983 K in the same mixture.*

**Pellet Size**	**Amount / g**	**Flow Rate / sccm**	**H_2O Formation Rate / μmol g_{cat}^{-1} s^{-1}**
125-177 μm	0.0191	65	4.5 ± 0.2
177-425 μm	0.0310	100	4.2 ± 0.2

The table shows that the rates are identical within experimental error, indicating that pellet-scale transport gradients for H_2 combustion are negligible under experimental conditions.

S3.3. Sand loading tests

In Table S6, sand alone was shown to have negligible activity for H_2 or CH_4 activation in CH_4-H_2-O_2 mixtures. Measurement of rates at two different acid-washed sand loadings in the presence of Na_2WO_4 was necessary to validate that the sand diluent does not catalyze pathways involving reaction products, e.g. H_2O-mediated combustion. The data in Table S8 demonstrate that rates of H_2 combustion are identical within experimental error for 0.24 g vs. 0.48 g of acid-washed sand added to the 5 wt.% Na_2WO_4/SiO_2 catalyst, validating that sand is inert during selective hydrogen combustion at the experimental conditions reported.

Table S8: *H_2O formation STYs over 5 wt. % Na_2WO_4/SiO_2 at varying sand diluent loadings. Reaction conditions: 5 kPa CH_4, 5 kPa H_2, 1.25 kPa O_2, balance He, 1.67 cm^3 (STP) s^{-1}, 923 or 983 K. Catalyst: 0.0309 g Na_2WO_4/SiO_2 + 0.24 g sand or 0.0313 g Na_2WO_4/SiO_2 + 0.48 g sand.*

**Sand Loading**	**Temperature / K**	**H_2O STY / μmol $g_{Na2WO4/SiO2}^{-1}$ s^{-1}**
0.24 g	983	10.7 ± 0.1
0.48 g	983	10.9 ± 0.2
0.24 g	923	3.7 ± 0.2
0.48 g	923	4.0 ± 0.1

This experiment also demonstrates that radial thermal gradients are insignificant. The left-hand side of Eq. S2.13 is proportional to the volumetric rate of H_2 combustion R_b, which is in turn proportional to 1/(1+b), where b is the sand:Na_2WO_4/SiO_2 volume ratio. Doubling the sand loading

from 0.24 g to 0.48 g causes b to increase from 3 to 6, which in turn means that R_b decreases by a factor of 1.75. If radial thermal gradients were significant, $-r_{A,obs}$ would be expected to change significantly with near-twofold changes in R_b.

S4. Calculation of conversion, selectivity, and rate

H_2 combustion selectivity S_{H2} is defined by Eq. S4.1:

$$S_{H_2} = \frac{X_{H_2}}{X_{H_2}+X_{C_xH_y}} \quad \text{(S4.1)}$$

where X_i is the conversion of reactant i to combustion products.

The conversions are calculated from the formation rates of combustion products H_2O and CO_x (x = 1 or 2), using the stoichiometries given in equations S4.2 and S4.3:

$$H_2 + \frac{1}{2}O_2 \rightarrow H_2O \quad \text{(S4.2)}$$

$$C_mH_n + \left(\frac{mx}{2}+\frac{n}{4}\right)O_2 \rightarrow m\,CO_x + \frac{n}{2}H_2O \quad \text{(S4.3)}$$

Thus, each mole of CO_x formed will result in n/2m moles of water being formed. The H_2 conversion is calculated assuming that H_2 combustion and C_mH_n combustion are the only significant sources of water, omitting the contributions of pathways forming other hydrocarbon products. The water from C_mH_n combustion is subtracted from the total water effluent flow rate, and this difference is divided by the water influent flow rate to give the H_2 conversion.

$$X_{H_2} = \frac{F_{H_2O,out} - n(F_{CO,out}+F_{CO_2,out})/2m}{F_{H_2,in}} \quad \text{(S4.4)}$$

C_mH_n conversion is calculated using the total formation rate of CO_x products.

$$X_{C_mH_n} = \frac{(F_{CO,out}+F_{CO_2,out})/n}{F_{C_mH_n,in}} \quad \text{(S4.5)}$$

where $F_{i,in}$ is the influent flow rate of reactant i and $F_{j,out}$ is the effluent flow rate of product j.

Combustion rates on a per-mass basis, $-r_i$, are computed by dividing the product effluent flow rates by the catalyst mass m_{cat}. Again, the water formed from C_mH_n combustion is deduced from CO_x formation rate and is subtracted from total water formation rate to compute the water formed from H_2 combustion.

$$-r_{H_2} = \frac{F_{H_2O,out} - n(F_{CO,out}+F_{CO_2,out})/2m}{m_{cat}} \quad \text{(S4.6)}$$

$$-r_{C_mH_n} = \frac{(F_{CO,out}+F_{CO_2,out})/n}{m_{cat}} \quad \text{(S4.7)}$$

At 923 K, hydrocarbon product formation rates are <0.5% of water formation rates in H_2-C_mH_n-O_2 (4:4:1) mixtures for C_mH_n = CH_4, C_2H_4, and C_6H_6. Hydrocarbon product formation rates in H_2-C_3H_6-O_2 (4:4:1) and H_2-C_2H_6-O_2 (4:4:1) and mixtures are ca. 2% and ca. 4% of H_2O formation rates, respectively. Thus, for all hydrocarbons, estimating H_2 conversion and combustion rate from H_2O and CO_x formation alone does not result in significant error.

S5. SHC experiments over NaO_x/SiO_2 and WO_3

S5.1. NaO_x/SiO_2 SHC experiments

0.8 wt. % NaO_x/SiO_2 was prepared following the same method and with the same nominal Na loading as 5 wt. % Na_2WO_4/SiO_2. 0.263 g NaO_x/SiO_2 was loaded without sand diluent and held in a CH_4-H_2-O_2 mixture (5 kPa CH_4, 5 kPa H_2, 1.25 kPa O_2, 1.67 cm^3 (STP) s^{-1}) for 160 ks. H_2O space-time yields (STYs) are shown in Fig. S2.

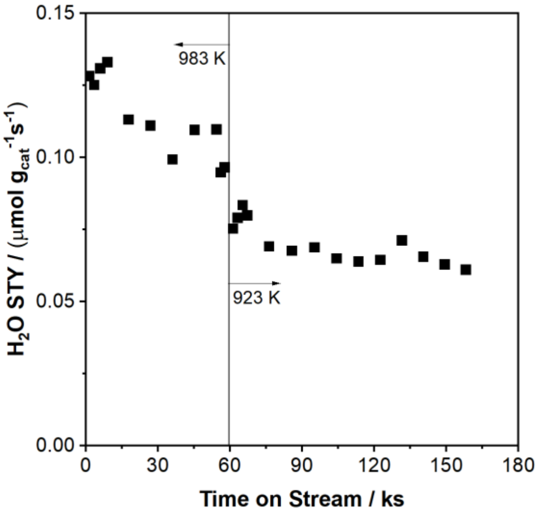

Figure S2: *H_2O formation space-time yield (STY) as a function of time on stream during SHC in CH_4-H_2-O_2 mixtures over a 0.8 wt. % NaO_x/SiO_2 catalyst. CO_x or C_2 products did not form in measurable quantities at any time. Reaction conditions: 5 kPa CH_4, 5 kPa H_2, 1.3 kPa O_2, balance He + N_2, 1.67 cm^3(STP) s^{-1} total flow rate, 923 K, 0.263 g NaO_x/SiO_2, undiluted.*

Note that H_2O effluent flow rates were comparable to those measured in the diluent-only bed at otherwise identical conditions (e.g., 0.022 µmol s^{-1} for NaO_x/SiO_2 versus 0.005 µmol s^{-1} for sand at 923 K). The water flow rate from the experiment using only sand from Table S6 was subtracted from the total measured water flow rate to obtain the STYs in Fig. S2. The rate is 0.11 µmol g_{cat} s^{-1} after 50 ks at 983 K and 0.064 µmol g_{cat}^{-1} s^{-1} after 50 ks at 923 K. Compared with rates measured over Na_2WO_4/SiO_2, rates measured over Na/SiO_2 are 120× and 70× lower at 983 K and 923 K, respectively.

S5.2. WO_3 SHC experiments

0.0391 g of WO_3 powder was physically mixed with 0.25 g sand for SHC experiments involving CH_4-H_2-O_2 (5 kPa CH_4, 5 kPa H_2, 1.25 kPa O_2, 1.67 cm^3 (STP) s^{-1} total flow rate) or C_3H_6-H_2-O_2 mixtures (5 kPa C_3H_6, 5 kPa H_2, 1.25 kPa O_2, 3.33 cm^3 (STP) s^{-1} total flow rate).

Apparent activation barriers for H_2 combustion in CH_4-H_2-O_2 mixtures are similar for WO_3 (104 ± 4 kJ mol^{-1}) compared with Na_2WO_4/SiO_2 (115 ± 5 kJ mol^{-1}), and no significant CH_4 activation

was observed. An Arrhenius plot for WO_3 is shown in Fig. S3. At 923 K, H_2O formation rates are 0.79 µmol g_{cat}^{-1} s^{-1} (0.18 µmol $mmol_W^{-1}$ s^{-1}) for WO_3 and 4.5 µmol g_{cat}^{-1} s^{-1} (26.5 µmol $mmol_W^{-1}$ s^{-1}) for Na_2WO_4/SiO_2.

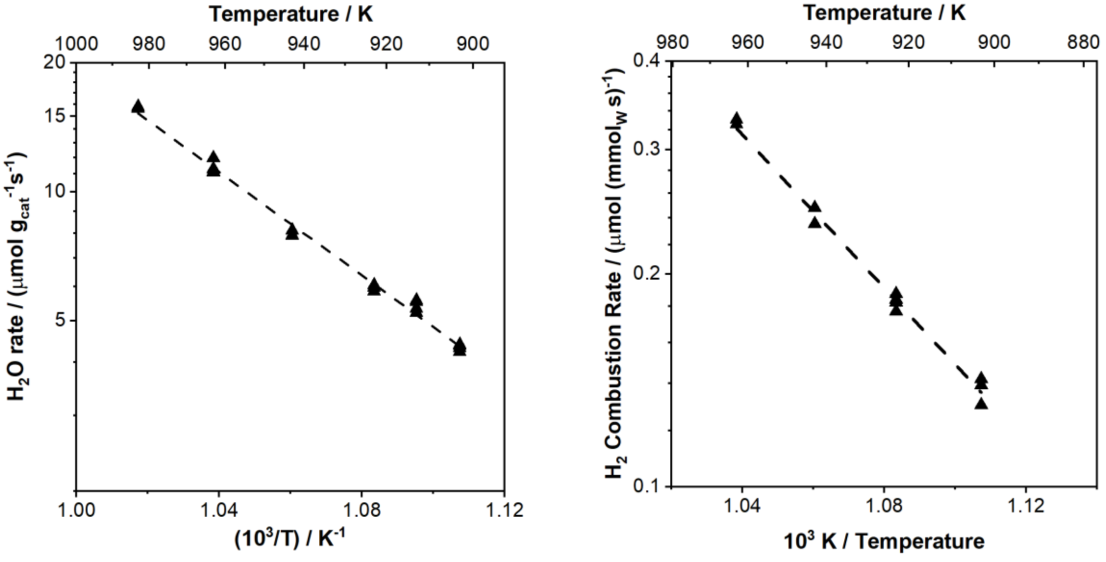

***Figure S3**: (a) Arrhenius plot for H_2 combustion rates in O_2 as a function of temperature over 5 wt. % Na_2WO_4/SiO_2 (5 kPa CH_4, 5 kPa H_2, 1.25 kPa O_2, balance He, 3.33 cm^3 (STP) s^{-1}, 903-993 K. Catalyst: 0.0290 g of Na_2WO_4/SiO_2). The calculated E_{app} is 115 ± 5 kJ mol^{-1}. (b) Arrhenius plot for H_2 combustion rates in O_2 as a function of temperature over pure WO_3. Reaction conditions: 5 kPa CH_4, 5 kPa H_2, 1.25 kPa O_2, balance He, 0.83 cm^3 (STP) s^{-1}, 893-983 K. Catalyst: 0.0391 g of WO_3 + 0.25 g acid-washed sand. The calculated E_{app} is 104 ± 4 kJ mol^{-1}.*

Combustion rates of C_3H_6 and H_2 are compared at 903 K in Table S9. The H_2 combustion rate in H_2-CH_4-O_2 mixtures is significantly lower than the combustion rate in H_2-C_3H_6-O_2 mixtures which would be estimated from the water formation rate using Eq. S4.4; however, other products (C_2, C_4-C_6) which are also associated with H_2O formation formed in significant quantities.

***Table S9**: H_2O and CO_x STYs measured when cofeeding different H_2-hydrocarbon-O_2 mixtures over pure WO_3. Reaction conditions: 5 kPa H_2, 5 kPa CH_4 or C_3H_6, 1.25 kPa O_2, 903 K, 1.67 or 3.33 cm^3 (STP) s^{-1} total flow rate. Catalyst bed: 0.0391 g WO_3 + 0.25 g acid-washed sand.*

Mixture	H_2O STY / µmol $mmol_W^{-1}$ s^{-1}	CO STY / µmol $mmol_W^{-1}$ s^{-1}	CO_2 STY / µmol $mmol_W^{-1}$ s^{-1}	X_{O2}
CH_4-H_2-O_2	0.14 ± 0.02	~0	~0	0.018
C_3H_6-H_2-O_2	1.7 ± 0.2	0.61 ± 0.05	0.39 ± 0.02	0.20

If the H_2 combustion rate in the C_3H_6-H_2-O_2 is assumed to be identical to that in the CH_4-H_2-O_2 mixture, the calculated hydrogen combustion selectivity is 0.30. WO_3 is not selective for

combustion of hydrogen over propylene at 903 K, unlike Na_2WO_4/SiO_2, suggesting that the alkali component is required for SHC.

S5.3. Li/MgO SHC Experiments

Li/MgO is another known OCM catalyst[12,13] containing an alkali metal-based material dispersed on an irreducible support, and thus belongs to a similar class of materials as Na_2WO_4/SiO_2. Li/MgO was also demonstrated to combust hydrogen preferentially over the hydrocarbons shown in Fig. S4.

For comparison, the measured hydrogen combustion selectivity over pure MgO at 873 K was 0.66 in C_2H_6-H_2-O_2 mixtures and 0.78 in CH_4-H_2-O_2 mixtures, lower than the values measured for Li/MgO (0.84 in C_2H_6-H_2-O_2 mixtures and 0.92 in CH_4-H_2-O_2 mixtures) under otherwise identical conditions. These results support the proposal that alkali metals facilitate the formation of H_2-selective oxygen species.

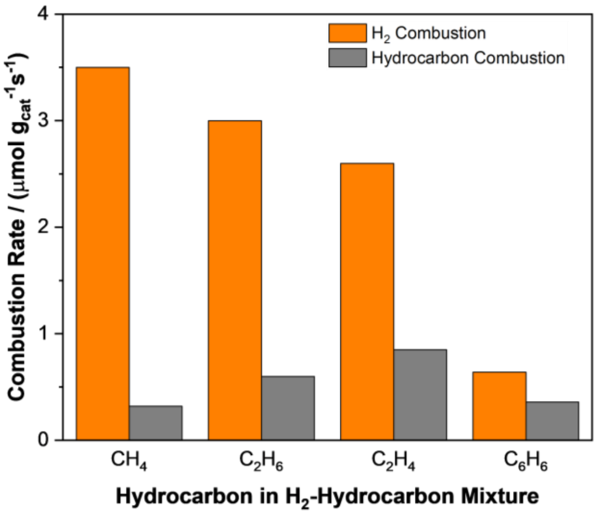

***Figure S4**: Combustion rates measured over Li/MgO for mixtures of H_2 and different hydrocarbons with cofed O_2. Reaction conditions: 5 kPa H_2, 5 kPa hydrocarbon, 0.5 kPa O_2, 873 K balance He, 1.67 cm^3 s^{-1} (STP). Catalyst bed: 0.0250 g Li/MgO + 0.25 g acid-washed sand.*

S6. Kinetic and isotopic experiments over Na_2WO_4/SiO_2

S6.1. H_2 and C_2H_4 combustion selectivity over changes in O_2 pressure

In addition to validating that SHC can occur over a broad range of C_2H_4:H_2 ratios at fixed O_2 partial pressure (Fig. 2b), experiments varying O_2 partial pressure in equimolar C_2H_4:H_2 feeds (5 or 15 kPa H_2, C_2H_4) demonstrate that SHC can occur over order-of-magnitude changes in O_2 pressure. Combustion selectivities are ≥96.5% at all conditions, as shown in Fig. S5.

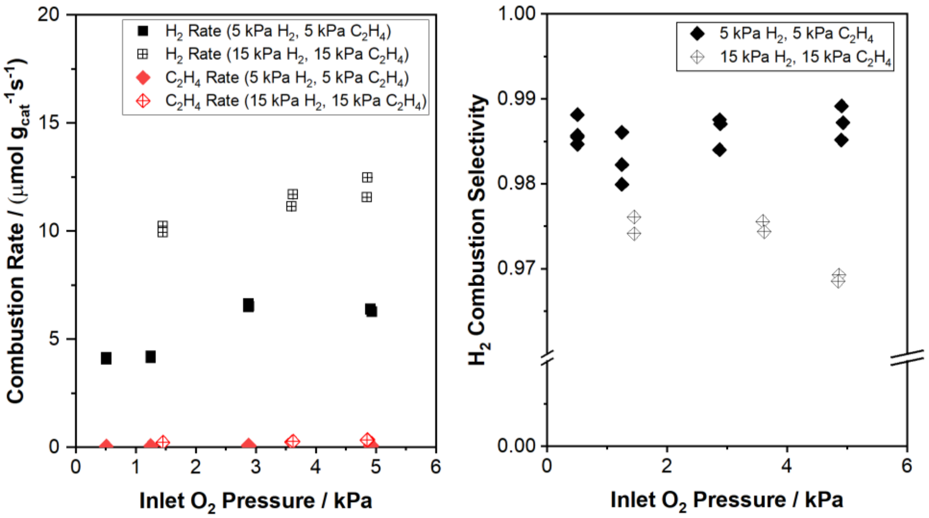

Figure S5 *(a) Rates of C_2H_4 and H_2 combustion and (b) combustion selectivities at varying O_2 partial pressures during SHC in C_2H_4-H_2-O_2 mixtures over a 5 wt. % Na_2WO_4/SiO_2 catalyst. Reaction conditions: 5 or 15 kPa C_2H_4, 5 or 15 kPa H_2, 1.3 kPa O_2, balance He + N_2, 1.67 cm^3(STP) s^{-1} total flow rate, 923 K, 0.0290 g Na_2WO_4/SiO_2.*

S6.2. Invariance of H_2 combustion rates with contact time

Contact time experiments were used to determine if reaction products influence H_2 combustion kinetics. H_2-C_2H_4-O_2 mixtures (5 kPa H_2, 5 kPa C_2H_4, 1.25 kPa O_2, balance N_2 + He) were introduced at varying total flow rates to attain different H_2 conversions and H_2O effluent partial pressures in the same catalyst bed. Fig. S6 demonstrates that H_2 combustion STYs are invariant for contact times ranging from 0.21-1.2 × 10^3 g_{cat} s mol^{-1} (4-20% O_2 conversion). H_2 combustion selectivity was >97.5% at all O_2 conversions and was did not change significantly with increasing O_2 conversion."

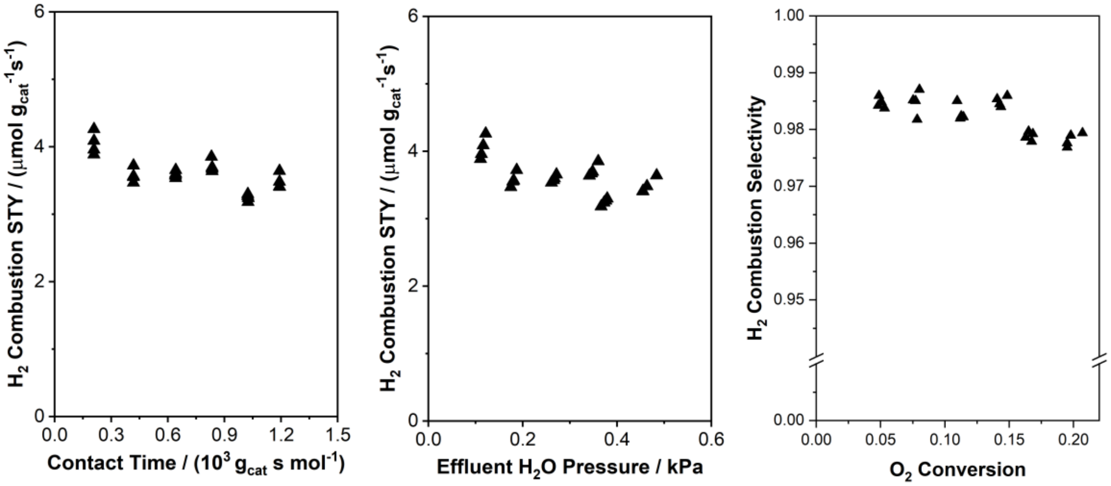

Figure S6*: (a) Space-time yields associated with H_2 combustion at contact times between 0.22 - 1.25×10^3 g_{cat} s mol^{-1}. (b) Space-time yields plotted as a function of effluent H_2O partial pressure for the same experiment. (c) H_2 combustion selectivity plotted as a function of O_2 conversion for the same experiment (5 kPa H_2, 5 kPa C_2H_4, 1.25 kPa O_2, balance He + N_2, 923 K, 0.58-3.33 cm^3 (STP) s^{-1} total flow rate).*

From these data, we surmise that H_2O does not significantly affect H_2 combustion kinetics at the partial pressures typical to the kinetic experiments herein.

S6.3. $^{16}O_2$-$^{18}O_2$ isotopic scrambling experiments

Experiments flowing $^{16}O_2$/$^{18}O_2$ mixtures over Na_2WO_4/SiO_2 and tracking the appearance of $^{16}O^{18}O$ via online mass spectrometry were performed to determine whether O-O bond activation is reversible and quasi-equilibrated, as proposed for $Mn/Na_2WO_4/SiO_2$ catalysts during OCM.[14] $^{16}O^{18}O$ formation was found to be negligible at 923 K in the presence of 0.6 kPa $^{16}O_2$ and 0.6 kPa $^{18}O_2$ whether H_2 is present or absent, as demonstrated in Fig. 3.

For O_2 dissociation to be quasi-equilibrated, the forward and reverse rates of O_2 dissociation and thus the rate of recombination of O* species to form $^{16}O^{18}O$ would each need to be significantly larger than the rate of O* removal by H_2 to form H_2O. H_2O effluent flow rates (0.14 μmol s^{-1}) at the conditions in Fig. 3 significantly exceed $^{16}O^{18}O$ effluent flow rates (<0.01 μmol s^{-1}); time-averaged $^{16}O^{18}O$ signals measured via online MS were identical whether bypassing or flowing over the catalyst. From these data, we infer that O_2 dissociation is not quasi-equilibrated. Instead, there are two possibilities:

(a) O_2 dissociation occurs but is irreversible, such that if $^{16}O_2$ and $^{18}O_2$ dissociate (to form 2 $^{16}O^*$ or 2 $^{18}O^*$), these species are strongly bound and cannot recombinatively desorb ($^{16}O^* + {}^{18}O^* \rightarrow {}^{16}O^{18}O + 2\,*$) to form $^{16}O^{18}O$.

(b) Hydrogen is required to remove one oxygen from an undissociated O_2* species while direct, unassisted O_2 dissociative adsorption ($O_2 + 2 * \rightarrow 2 O^*$) does not occur at all under our process conditions.

The reversibility z of the reaction $^{16}O_2 + {}^{18}O_2 \leftrightarrow 2\ {}^{16}O^{18}O$ is given by Eq. S6.1:

$$z = \left(\frac{P^2_{^{16}O^{18}O}}{P_{^{16}O_2} P_{^{18}O_2}} \right) / K_{ex} \quad \text{(S6.1)}$$

Here, the equilibrium constant is $K_{ex} = 4$, corresponding to complete scrambling of O_2 isotopologues ($^{16}O_2$:$^{16}O^{18}O$:$^{18}O_2$ = 1:2:1). An upper bound on z, calculated from the time-averaged value of z between 0-0.46 ks, is 2×10^{-4}.

S7. Characterization of Na_2WO_4/SiO_2

S7.1. Ex situ XRD for pre- and post-reaction samples

Fig. S7 shows XRD patterns obtained before and after SHC reactions for Na_2WO_4/SiO_2 catalysts. Crystallographic information was obtained from the Materials Project database[15] and patterns were calculated using VESTA software. The post-reaction sample was held at elevated temperature (≥923 K) in an SHC mixture (5 kPa CH_4, 5 kPa H_2, 1.25 kPa O_2) for >16 h before removal.

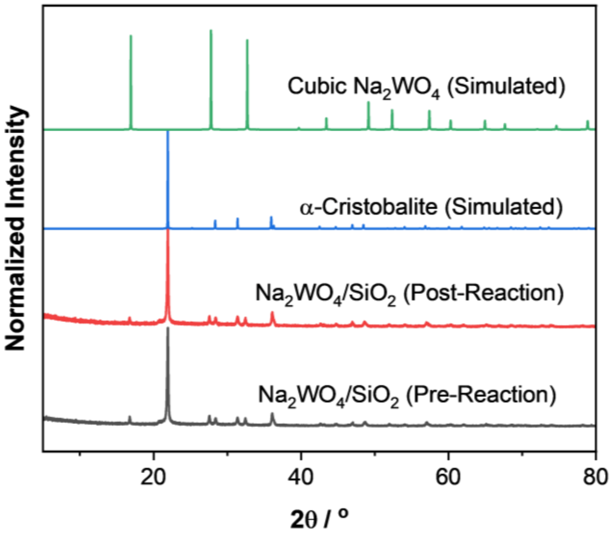

***Figure S7**: Ex situ XRD patterns for pre- and post-reaction Na_2WO_4/SiO_2 samples, with cubic Na_2WO_4 and α-cristobalite simulated patterns for comparison.*

S7.2. High temperature XRD patterns for Na_2WO_4/SiO_2 in air

Diffraction patterns were obtained for the Na_2WO_4/SiO_2 catalyst in the 298-983 K range, with the sample heated at 0.167 K s^{-1} between acquisition of powder XRD patterns. After the 983 K measurement, a diffraction pattern was obtained at 923 K following a -0.167 K s^{-1} cooling step. The time to acquire each pattern was 1250 s. Results are shown in Fig. S8. α-cristobalite (space group: $P4_12_12$) and cubic Na_2WO_4 (space group: Fd-3m) were observed at ambient temperature, while β-cristobalite (space group: Fd-3m) and cubic Na_2WO_4 were observed at 773 K $\leq$ T $\leq$ 948 K.

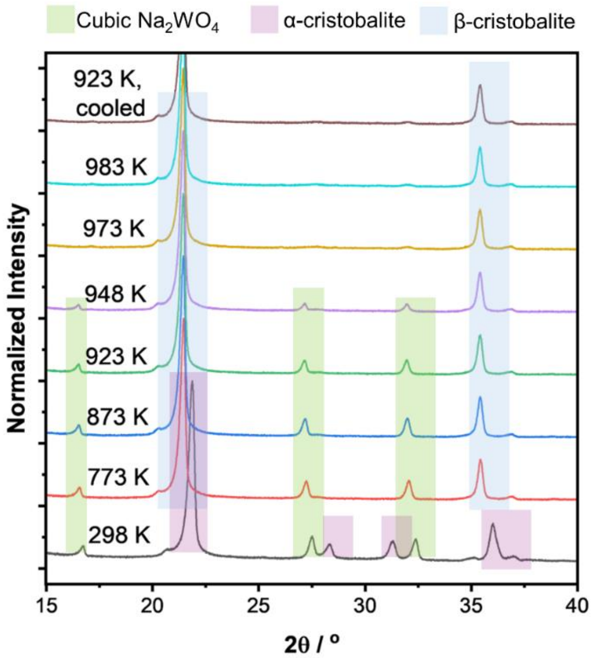

Figure S8: *XRD patterns (λ = 1.54 nm) obtained upon heating a 5 wt. % Na_2WO_4/SiO_2 catalyst in air to 973 K. Diffraction patterns were recorded from 2θ = 15-40°, with the detector moving in Δ2θ = 0.005° steps at a rate of 1.5° min^{-1}. The heating rate between measurements was 0.167 K s^{-1}. The catalyst bed was held under a graphite dome to facilitate in situ measurements.*

Structural features of crystalline Na_2WO_4 were noted to disappear at 973 K, a temperature exceeding the bulk melting point of Na_2WO_4. Crystalline features were not observed on the timescale of the experiment after cooling to 923 K.

S7.3. X-ray absorption spectroscopy at SHC temperatures

W L_{III}-edge XAS spectra were obtained in flowing He (0.33 cm^3 (STP) s^{-1}) at 298-973 K. Edge positions (E_0) for each spectrum were computed from the first maximum of the first derivative of μ(E) and are shown in Table S10. For comparison, the measured edge positions of the solid Na_2WO_4, WO_2, and W reference materials were 10209.6 eV, 10208.0 eV, and 10207.0 eV, respectively. The edge positions for all spectra are identical within error to the edge position of the Na_2WO_4 reference material (10209.6 eV), consistent with a mean tungsten oxidation state of +6 at all temperatures.

χ(k) spectra for six representative measurements (298 K, 573 K, 773 K, 873 K, 923 K, 983 K) are given below in Fig. S9. The spectra for all measurements obtained between 873-983 K are qualitatively identical.

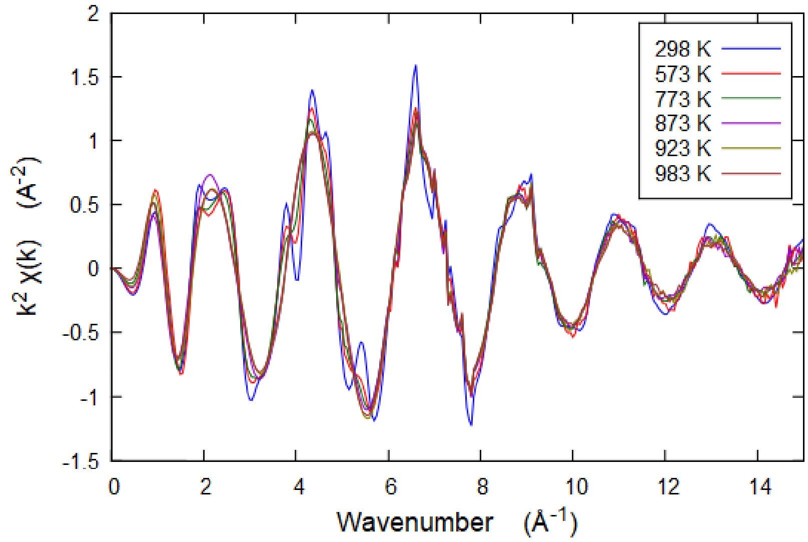

***Figure S9**: Comparison of W L_{III}-edge k^2-weighted χ(k) spectra for Na_2WO_4/SiO_2 samples held in He at varying temperatures.*

Referenced to the crystal structure of cubic Na_2WO_4, the EXAFS parameters for the W-O scattering path necessary to achieve convergence are also given in Table S10, and a sample EXAFS fit for the 923 K measurement is shown in Fig. S10. First-shell fits were performed with k-weights of 1, 2, and 3 in the range 3.8 $Å^{-1} \leq k \leq 13.7$ $Å^{-1}$ with 1.0 Å $\leq r \leq 2.1$ Å and a Hanning window with dk = 1.0 Å.

Table S10: *XANES edge positions and EXAFS fitting results for Na_2WO_4/SiO_2 samples held in He at varying temperatures. The fits involve a single W-O scattering path which describes the first coordination shell. For reference, the W-O bond length for crystalline Na_2WO_4 is 1.783 Å.*

**Temperature / K**	**Edge Position / eV**	**Coordination Number (W-O)**	**Interatomic Distance / Å**	**Debye-Waller Factor / Å^2**
298	10209.6 ± 0.2	3.3 ± 0.4	1.78 ± 0.01	0.0011 ± 0.0008
773	10209.8 ± 0.2	3.4 ± 0.4	1.78 ± 0.01	0.0019 ± 0.0010
873	10209.8 ± 0.2	3.5 ± 0.5	1.78 ± 0.01	0.0021 ± 0.0011
923	10209.5 ± 0.2	3.5 ± 0.5	1.78 ± 0.01	0.0022 ± 0.0010
953	10209.7 ± 0.2	3.6 ± 0.5	1.78 ± 0.01	0.0024 ± 0.0011
983	10209.8 ± 0.2	3.7 ± 0.4	1.78 ± 0.01	0.0026 ± 0.0010
923†	10209.7 ± 0.2	3.5 ± 0.4	1.78 ± 0.01	0.0021 ± 0.0010
298‡	10209.4 ± 0.2	3.5 ± 0.4	1.78 ± 0.01	0.0011 ± 0.0009

†*After cooling from 983 K.*
‡*After rapidly cooling from 873 K*

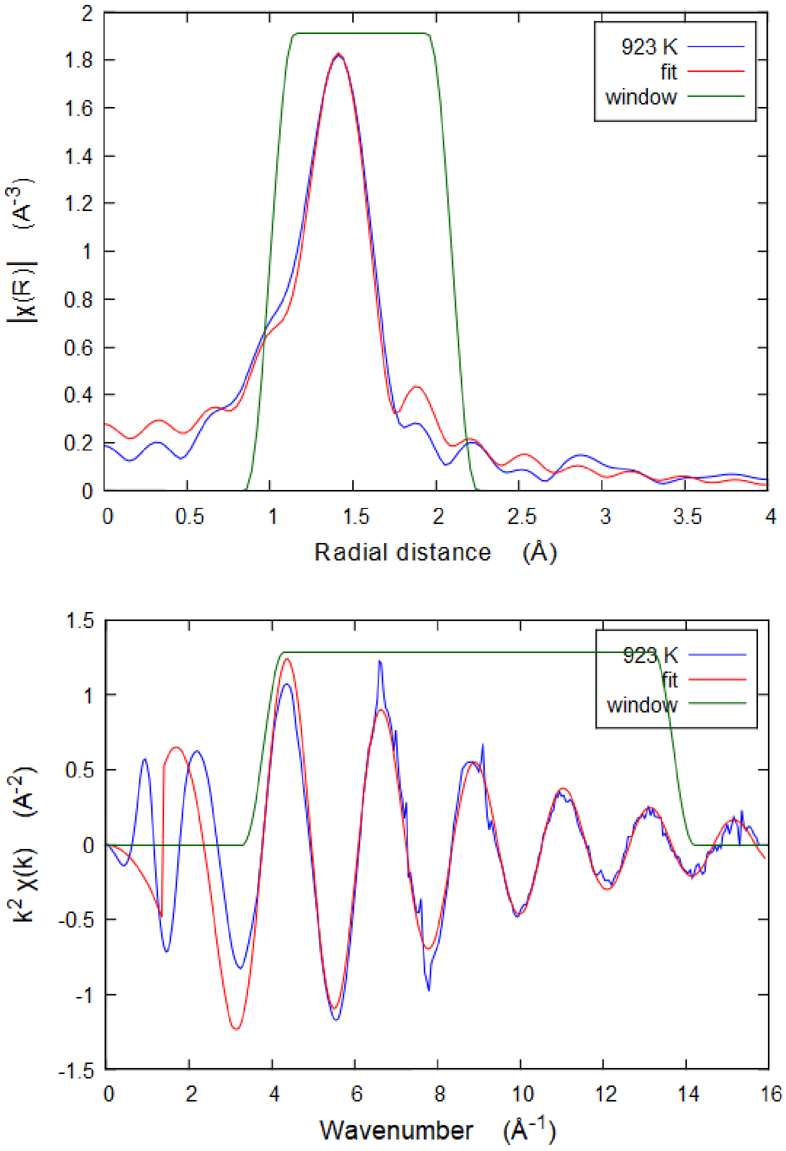

Figure S10: *W L_{III}-edge (a) $|\chi(R)|$ and (b) k^2-weighted $\chi(k)$ spectra of the Na_2WO_4/SiO_2 sample held in He at 923 K. The FEFF fit (calculated using Artemis software[7]) is also shown. Other fits for $T \geq 873$ K are visually similar and give similar results for coordination number and other fitting parameters, consistent with the similar $|\chi(R)|$ spectra shown in Fig. 4 between 873-983 K.*

Following 873 K measurements, the sample was rapidly cooled (ca. 0.5 K s^{-1}) from 873 K to 298 K. After this rapid cooling step, X-ray absorption spectra were measured; the temperature was subsequently increased at 0.083 K s^{-1} to obtain 923 K and 983 K spectra. The 873 K spectrum, 983 K spectrum, and 298 K |χ(R)| spectra obtained before and after heating the sample to 873 K are overlaid in Fig. S11; the lack of a second-shell feature at 873 K persists upon rapid cooling to 298 K. This comparison demonstrates that the loss of second-shell features at elevated temperatures below the bulk Na_2WO_4 melting point is related to the structural transformation of the catalyst and is not caused by thermal disorder alone.

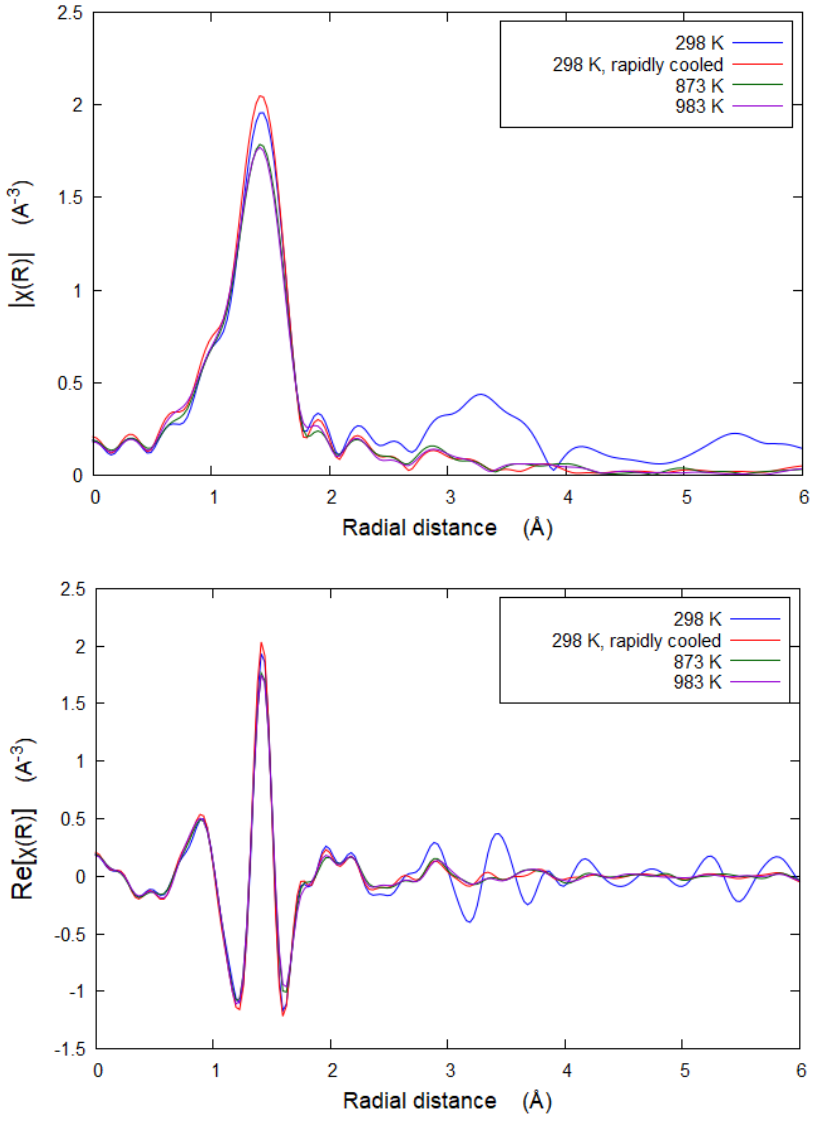

Figure S11: *Comparison of W L_{III}-edge k^2-weighted (a)|χ(R)|, and (b) Re(χ(R)) spectra for Na_2WO_4/SiO_2 samples held in He at varying temperatures.*

References

[1] D. Kiani, S. Sourav, I. E. Wachs, J. Baltrusaitis, *Catal. Sci. Technol.* **2020**, *10*, 3334–3345.

[2] S. Sourav, Y. Wang, D. Kiani, J. Baltrusaitis, R. R. Fushimi, I. E. Wachs, *Angewandte Chemie International Edition* **2021**, *60*, 21502–21511.

[3] D. E. Mears, *Ind. Eng. Chem. Proc. Des. Dev.* **1971**, *10*, 541–547.

[4] O. Proux, **2021**, 10 spectra.

[5] S. El Mohammad, O. Proux, A. Aguilar, J.-L. Hazemann, C. Legens, C. Chizallet, K. Larmier, *Inorg. Chem.* **2023**, *62*, 7545–7556.

[6] B. Schmitt, P. Bollard, D. Albert, A. Garenne, M. Gorbacheva, L. Bonal, M. Furrer, P. Volcke, the S. P. C. and, **2017**, DOI 10.26302/SSHADE.

[7] B. Ravel, M. Newville, *J Synchrotron Rad* **2005**, *12*, 537–541.

[8] E. N. Fuller, P. D. Schettler, J. Calvin. Giddings, *Ind. Eng. Chem.* **1966**, *58*, 18–27.

[9] H. Petersen, *The Properties of Helium: Density, Specific Heats, Viscosity, and Thermal Conductivity at Pressures from 1 to 100 Bar and from Room Temperature to about 1800 K*, Risoe National Laboratory, Denmark, **1970**.

[10] D. A. Hickman, J. C. Degenstein, F. H. Ribeiro, *Current Opinion in Chemical Engineering* **2016**, *13*, 1–9.

[11] A. M. Hofmeister, *The Canadian Mineralogist* **2013**, *51*, 705–714.

[12] T. Ito, J. Wang, C. H. Lin, J. H. Lunsford, *J. Am. Chem. Soc.* **1985**, *107*, 5062–5068.

[13] J. H. Lunsford, *Angewandte Chemie International Edition in English* **1995**, *34*, 970–980.

[14] K. Takanabe, E. Iglesia, *J. Phys. Chem. C* **2009**, *113*, 10131–10145.

[15] A. Jain, S. P. Ong, G. Hautier, W. Chen, W. D. Richards, S. Dacek, S. Cholia, D. Gunter, D. Skinner, G. Ceder, K. A. Persson, *APL Materials* **2013**, *1*, 011002.
